# Safranal Enhances the Efficacy of Praziquantel Against *Schistosoma mansoni* Infection and Alleviates Liver Fibrosis, Inflammation and Oxidative Stress in Mice

**DOI:** 10.3390/jox16040120

**Published:** 2026-06-26

**Authors:** Azza Fahmy, Amany Mohammed Mohmmed Hegab, Hanan S. Mossalem, Samah Sulaiman Abuzahrah, Saud Omar Alafghani, Alaaeldin Ahmed Hamza, Nouf Juaid, Amr Amin

**Affiliations:** 1 Departments of Parasitology, Immunology & Drug Evaluation, Theodor Bilharz Research Institute, Giza 12411, Egypt; azzafhmy@gmail.com; 2 Developmental Pharmacology and Acute Toxicity Department, Egyptian Drug Authority (EDA), Formerly National Organization of Drug Control and Research, Giza 12112, Egypt; manalhegab2630@gmail.com; 3 Medical Malacology Department, Theodor Bilharz Research Institute, Giza 12411, Egypt; hanan.mossalem@yahoo.com; 4 Department of Biological Sciences, College of Science, University of Jeddah, Jeddah 21589, Saudi Arabia; sabozahera@uj.edu.sa; 5 Department of Histopathology, Al Hada Military Hospital, Taif 26792, Saudi Arabia; soalafghani@gmail.com; 6 Biology Department, Egyptian Drug Authority (EDA), Formerly National Organization of Drug Control and Research (NODCAR), Giza 12611, Egypt; 7 Department of Clinical Laboratory Science, Inaya Medical College, Riyadh 12211, Saudi Arabia; 8 Department of Basic Medical Sciences, College of Medicine, University of Sharjah, Sharjah 27272, United Arab Emirates; a.amin@sharjah.ac.ae

**Keywords:** *Schistosoma mansoni*, safranal, experimental murine model, antifibrotic therapy, antioxidant, anti-inflammatory effects

## Abstract

Although praziquantel (PZQ) is the main antischistosomal drug currently in use, concerns remain regarding incomplete reversal of schistosomiasis-induced pathology and the emergence of drug resistance. This study evaluates the combined effect of PZQ with safranal, a bioactive saffron constituent, on *Schistosoma mansoni*-induced pathology in mice. Male CD1 Swiss albino mice were exposed to 60 *S. mansoni* cercariae and, at week 9 post-infection, were treated with PZQ (500 mg/kg orally for two consecutive days), safranal (50 mg/kg/day), or both, for three weeks. The animals were sacrificed at week 11 post-infection. Worm and egg burdens, liver histopathology, fibrotic markers, oxidative stress, and inflammatory cytokines were assessed. Combined PZQ + safranal therapy significantly reduced adult worm counts and hepatic and intestinal egg loads compared to PZQ alone. All treatments decreased liver index (hepatomegaly), with the combination treatment providing the best intervention. Histological analyses revealed significantly reduced granuloma size and hepatic necrosis post-treatment, particularly in the combination group. The levels of proinflammatory cytokines (TNF-α, IL-1β) and Th2 cytokines (IL-4, IL-5, IL-6, IL-10) were significantly lowered in treated mice, most notably with the combination treatment. Oxidative stress was also markedly attenuated, and infected mice exhibited elevated malondialdehyde and depleted antioxidant enzymes (SOD, CAT, GSH). Interestingly, PZQ and/or safranal restored antioxidant status and reduced lipid peroxidation, with the combination being most effective. Furthermore, collagen deposition and expression of hepatic fibrotic markers α-smooth muscle actin (α-SMA), TGF-β1, and matrix metalloproteinase-9 were most effectively suppressed by combined therapy. To conclude, safranal enhances PZQ’s antischistosomal efficacy and confers additive protection against *Schistosoma*-induced liver fibrosis.

## 1. Introduction

Schistosomiasis, also referred to as bilharzia, is an infectious disease affecting both humans and animals, caused by trematode flatworms belonging to the genus *Schistosoma* [1]. It is recognized as a prevalent neglected tropical disease in numerous developing nations [2]. Infections of *Schistosoma mansoni* (*S. mansoni*), *S. haematobium*, or *S. japonicum* collectively impact approximately 230 million individuals globally, with up to 700 million still vulnerable to infection, leading to an estimated 200,000 annual fatalities [3,4]. The majority of those affected reside in Sub-Saharan Africa, the Middle East, South America, and Southeast Asia [2,4]. Rather than being expelled, most of the adult worm’s eggs become trapped in the parasite’s organs. Specifically, in *S. mansoni* infection, this occurs in the liver, provoking immune responses and inflammation, potentially leading to granuloma formation and liver fibrosis [1]. Granulomas are primarily induced by chronic antigenic stimulation and cytokine-driven recruitment and activation, involving various immune cells such as lymphocytes, eosinophils, and macrophages, which secrete inflammatory cytokines like IL-1, IL-2, TNF-, and T helper 2 (Th2) cytokines such as IL-4, IL-5, IL-6, IL-9, and IL-13 [5]. Granulomas play a crucial role in the pathogenesis of schistosomiasis, as collagen deposition and fibrosis development lead to fibro-obstructive disease [1].Liver fibrosis is characterized by the activation of hepatic stellate cells (HSCs) that transdifferentiate into myofibroblast-like cells, which are the major source of excessive extracellular matrix (ECM) [6]. The activation of HSCs occurs in three stages: initiation, perpetuation, and resolution. Although TGF-β serves as a key mediator in liver fibrosis by activating HSCs and fibroblasts to produce ECM proteins, thus contributing to liver fibrosis, IL-13 acts as a key contributor to hepatic fibrogenesis, particularly in Th2-biased conditions such as schistosomiasis [7].

The primary treatment for schistosomiasis is praziquantel (PZQ), which effectively eliminates the worm. It does not prevent further infections or treat liver fibrosis [8]. Additionally, there are concerns about PZQ resistance supported by laboratory evidence demonstrated in vivo and in vitro studies [9]. Due to the inability of PZQ to reverse organ damage caused by schistosomiasis, treatment efficacy is often limited [10,11]. Consequently, there is a critical need for novel and effective treatments for *schistosomiasis,* with combination therapies involving PZQ and natural products carrying unique promise. Various natural products, including *Ziziphus spina-christi*, Ozoroa pulcherrima Schweinf, schisandrin B, *Balanities aegyptiaca*, Juglone, wogonin, and casticin, have demonstrated effective antifibrotic and antiparasitic properties [11,12,13,14,15]. Saffron, either alone or in combination with PZQ, improved the host’s antioxidant and immunological status or accelerated the healing of the pathological granulomatous lesions of the liver architecture in the *S. mansoni* mouse model [16]. Safranal, a major component of the spice saffron (*Crocus sativus*), has attracted interest due to its diverse bioactivities, including potent antioxidant and anti-inflammatory effects [17,18,19]. Safranal has demonstrated various therapeutic effects, including anti-inflammatory, antidepressant, anxiolytic, anti-asthmatic, anti-hypertensive, anticonvulsant, anticancer, and antigenotoxic properties in animal and cellular models [19,20,21,22]. Its potent ability to scavenge free radicals and suppress inflammatory cytokines, together with its favorable safety profile demonstrated in preclinical studies at low to moderate doses, makes safranal an excellent candidate to mitigate liver injury [19,23]. Therefore, we chose safranal as a specified single constituent of saffron in the current investigation to reduce the variability associated with crude plant extracts and more accurately assess its potential as an adjuvant to PZQ in *S. mansoni* infection.

The murine model of *S. mansoni* infection is well-established for studying schistosomiasis-induced liver fibrosis and for testing antifibrotic interventions [24]. In this study, we hypothesize that co-administration of safranal with PZQ enhances the therapeutic efficacy against *S. mansoni* and reduce the consequent liver fibrosis, inflammation, and oxidative stress. This investigation thus evaluates the effects of combined PZQ and safranal therapy on parasitological parameters, liver histopathology, fibrotic markers, oxidative stress indices, and inflammatory cytokines in a mouse model of chronic schistosomiasis.

## 2. Materials and Methods

### 2.1. Experimental Animals

The experiments used 5–8-week-old, fully developed male CD1 Swiss albino mice weighing 20–25 g. The mice were acquired from the animal house of the Egyptian Drug Authorization (EDA) and the Schistosome Biology Supply Core (SBSC) of the Theodor Bilharz Research Institute in Giza, Egypt. The research followed the criteria of the US National Institutes of Health’s *Guide for Care and Use of Laboratory Animals* (NIH Publication No. 8523, amended 2011). The animal ethics committee of NODCAR approved the animal care and handling (Approval number of the protocol: NODCAR/7/10/2024). The mice were housed in cages with unrestricted access to food and water and were supplemented with a standard commercial pelleted diet from El-Kahira, an Egyptian company specializing in oils and soap. The specimens were maintained in meticulously controlled laboratory settings with 12 h alternate periods of light and darkness, a consistent temperature of 25 ± 2 °C, and 50 ± 15% humidity.

### 2.2. Mice Infection

Infected snails of the *Biomphalaria alexandrina* species were used to acquire the Egyptian strain of *S. mansoni* cercariae from the Schistosome Biologic Supply Center at Theodor Bilharz Experimental Institute in Giza, Egypt. The snails were allowed to release their cercariae in completely dechlorinated water after being exposed to direct sunshine for one hour. The cercariae were enumerated in 0.1 mL of dechlorinated water using a dissecting microscope after adding a single droplet of Lugol’s reagent. To transmit the infection to the animals, each mouse was moved to a sterile sedimentation flask and submerged it caudally for 90 min in a small amount of dechlorinated water containing 60 ± 10 recently shed cercariae [25].

### 2.3. Drugs

Safranal (W338907 Aldrich) was obtained from Sigma-Aldrich (St. Louis, MO, USA). Praziquantel (PZQ) (Distocide^®^ was purchased from EPICO (Tenth of Ramadan City, Cairo, Egypt)).

### 2.4. Dosage Selection

Both safranal and PZQ were freshly suspended in 0.1% Tween/distilled water before administration via oral gavage using a stainless-steel oral cannula. PZQ was given at a dose of 500 mg/kg for two days at 9 weeks post-infection (PI) [15,26,27]. Safranal was given at a dose of 50 mg/kg/day. Safranal at 50 mg/kg/day was not toxic to the immune system in mice and had no toxicity on humoral and cellular immune responses [28]. It was also found to have anti-inflammatory and antioxidant properties in prior chemically induced inflammation and oxidative stress in mice and rats [29].

### 2.5. Experimental Design, Monitoring, and Humane Endpoints

**Figure 1:**
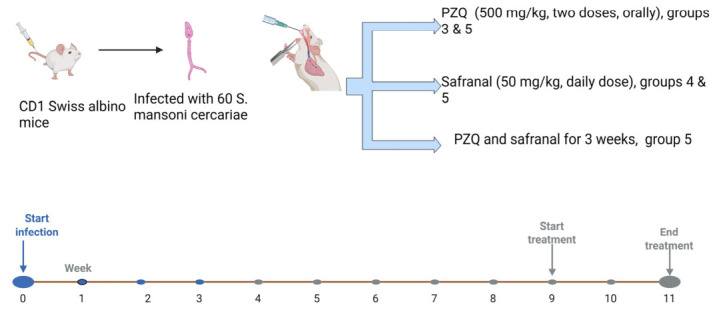
Experimental design and timeline followed during the present study.

Sixty mice were randomly divided into five groups (n = 12 per group): (1) Uninfected control (Normal); (2) Infected untreated (vehicle only); (3) Infected + PZQ; (4) Infected + safranal; (5) Infected + PZQ + safranal. Groups 2–5 were percutaneously infected with 60 cercariae as above. At 9 weeks post-infection, treatments were administered as follows: group 3 received PZQ (500 mg/kg) orally once daily for 2 days; group 4 received safranal (50 mg/kg) orally once daily for 3 weeks; group 5 received both PZQ (as in group 3) and safranal (as in group 4). Normal and infected-untreated groups received the vehicle (0.1% Tween/distilled water) by oral gavage. All administrations were given via a stainless steel oral cannula. The planned study duration was 11 weeks post-infection (i.e., the mice were euthanized three weeks after treatment initiation). Figure 1 illustrates the schematic representation of the experimental design and chronology employed in the current study.

Humane endpoints: Death was not used as an experimental endpoint. Prior to study initiation, humane endpoints were predefined, and any animal meeting the criteria was promptly euthanized. Criteria included: ≥20% loss of baseline body weight (or ≥15% within 48 h); body-condition score ≤ 2/5; persistent severe lethargy or recumbency; inability to access food/water; severe dehydration (skin tenting > 2 s); dyspnea/labored breathing unresponsive to brief handling; self-mutilation; unrelieved distress/pain; moribund state; or any condition judged by the attending veterinarian to indicate poor prognosis.

Monitoring: The animals were observed once daily (weeks 1–8) and twice daily during acute disease phases and the treatment window (weeks 9–11). Body weight and clinical scores were recorded 7 times per week. The animals approaching criteria triggered immediate review. Upon confirmation of criteria, euthanasia was performed without delay (generally within 30 min of detection and on the same day).

Welfare measures, anesthesia, and euthanasia: No surgical procedures were performed; routine analgesia was not indicated. Transient distress associated with gavage was minimized by trained handling and supportive care. For terminal procedures at 11 weeks post-infection or upon reaching endpoints, mice were anesthetized with sodium thiopental (100 mg/kg, i.p.) and euthanized by isoflurane overdose, followed by cervical dislocation in accordance with AVMA guidelines. Numbers and outcomes: A total of 60 animals were enrolled. There were zero death cases from advanced schistosomiasis-related complications. All unanticipated events have been reviewed by us and recorded.

### 2.6. Sample Collections

At 11 weeks post-infection, the mice were weighed and anesthetized with sodium thiopental (100 mg/kg, i.p.) prior to euthanasia. Blood was collected by cardiac puncture, and serum was separated by centrifugation 1850× *g* for 15 min, and the samples were stored at −80 °C for biochemical assays. Livers were excised and weighed to calculate the liver index (liver weight/body weight). Liver and intestine samples were collected and processed as described below. Six mice per group were randomly allocated to parasitological and histological analyses, and six mice per group were randomly used for biochemical, oxidative stress, and PCR analysis. Following the removal of the livers, a portion of the right lobe was isolated for PCR analysis. The remaining tissue was promptly fixed and subjected to histopathological and immunohistochemical (IHC) investigations. The left and caudate lobes were frozen and utilized to quantify cytokine levels and oxidative stress markers. Sample size was calculated using G*Power 3.1.9.7 software. With an effect size of 0.90 (based on preliminary data showing large differences between treatment groups), an α-error probability of 0.05, and power of 0.80, the minimum required sample size was determined to be 5 animals per group. We used 6 animals per group to account for potential attrition and to ensure adequate statistical power.

### 2.7. Worm Recovery and Egg Counting

Adult *S. mansoni* worms were recovered by perfusion of the hepatic portal system from six mice from each infected group [30]. Following euthanasia, each mouse’s abdominal cavity was exposed, and a cut was made at the portal vein. A needle connected to a perfusion pump was inserted into the abdominal aorta, and a significant volume of isotonic saline solution containing 1000 IU/L of heparin was delivered. The wash-through from each mouse was collected in a 250 mL beaker. The worms from each mouse were allowed to settle for approximately 20 min in a 20 cm glass Petri dish before being counted using a stereomicroscope (Olympus, CHK, Japan). For egg counts, a portion of liver or intestine was weighed and digested in 5% potassium hydroxide at 37 °C. The tissue digests were centrifuged at 370× *g* for 3 min at room temperature, washed, and eggs in a known aliquot (100 μL) were counted under a light microscope. Egg numbers were normalized to tissue weight and expressed as eggs per gram of tissue [31].

### 2.8. Histopathological Examination and Measurement of Mean Granuloma Diameter

Following perfusion, the right lobe of the liver was immersed in a 10% formaldehyde solution made in phosphate-buffered saline solvent. Once tissue samples had been dehydrated in consecutive ethanol (50–100%) and xylene baths, they were embedded in paraffin. Sections five micrometers in thickness were stained with hematoxylin and eosin (H&E) to qualitatively analyze liver damage and assess the inflammatory granulomatous response. The evaluation criteria for hepatic tissue were established according to the degree of liver injury, encompassing the presence of inflammatory cell infiltration, vacuolation, and necrosis of liver cells. Liver injury was scored on a 0–3 scale based on the extent of hepatocyte necrosis, inflammatory infiltration, and structural damage (0 = none; 1 = mild; 2 = moderate; 3 = severe) [32]. Quantification or scoring was performed on six slides in each group. In total, 10 random microscopic fields were examined on each slide. Granulomas containing a single egg were measured by microscopy: images were captured by a digital camera (DCM35; Scopotek^®^, Shenzhen, China) under a 10× objective and analyzed with Fiji ImageJ (2.16.0) software, http://imagej.net/software/fiji/ (accessed on 9 May 2026) (NIH, Bethesda, MD, USA). software to determine the granuloma diameter (six granulomas per mouse). The average granuloma diameter per group was calculated in micrometers (μm).

Collagen deposition was assessed by Sirius Red staining (SciTek kit). Stained sections were examined under polarized light. The collagen-positive area (red) was quantified using ImageJ in six non-overlapping fields (200× magnification) per section. Fibrotic area was expressed as a percentage of collagen-stained area relative to total tissue area. For quantitative image analysis, portal tracts and periportal fibrous septa were selected, while blood vessels and perivascular collagen were excluded from the regions of interest to avoid overestimation of collagen area.

### 2.9. Evaluation of Liver Damage and Oxidative Stress Markers

Quantification of blood indicators of liver damage, including alanine and aspartate aminotransferases (ALT and AST) and alkaline phosphatase (ALP), was conducted using commercially available kits from Bio Diagnostics in Egypt, according to the instructions provided by the vendor. To evaluate the oxidative stress indicators, liver tissue was homogenized in four volumes (*w/v*) of cold 0.1 M potassium phosphate buffer (pH 7.4) containing 1 mM EDTA. Subsequently, the mixture was subjected to centrifugation at 4 °C and 600× *g* for 10 min and then cooled by centrifugation at 10,000× *g* for 20 min. In accordance with the manufacturer’s instructions, the liquid component was collected and stored at a temperature of −80 °C for the purpose of analyzing reduced glutathione (GSH), superoxide dismutase (SOD), and lipid peroxidation via malondialdehyde production (MDA) in the liver. Glutathione (GSH) concentrations in liver homogenate were quantified using the Van Doorn et al. technique [33]. This approach determines the efficiency through the interaction between the thiol group of glutathione (GSH) at a pH of 8.0 and Ellman’s reagent, namely 5,5-dithiobis (2-nitrobenzoic acid). The presence of the 5-thiol-2-nitrobenzoate anion is verified by the production of a bright yellow hue upon contact. The analysis of MDA, a biomarker for lipid peroxidation, was performed quantitatively using the technique developed by Uchiyama and Mihara in 1978 [34]. This technology relies on the interaction between MDA and thiobarbituric acid (TBA), which leads to the formation of a pink crystal with the greatest light absorption at a wavelength of 535 nm. The Aebi technique [35] was employed in this study to quantify catalase (CAT) activity. The rapid reduction of H_2_O_2_ is quantified using this technique at a specific wavelength of 240 nm, and the recorded data is expressed in units per milligram of protein. The authors Nandi and Chatterjee [36] presented a comprehensive account of an experimental technique for measuring the level of superoxide dismutase (SOD) activity in liver homogenates. This strategy relies on the capacity of superoxide dismutase (SOD) to inhibit the oxidation of the enzyme pyrogallol at a pH of 8.5.

### 2.10. IHC Analysis for TGF-β1, α-SMA and NF-κB Proteins

To conduct immunohistochemistry analysis, 4 μm thick tissue sections were de-paraffinized using xylene and then hydrated in a graded ethanol solution. They were then rinsed with a 1 mM phosphate buffer at a pH of 6.0. For antigen retrieval, tissue sections were treated with 1% hydrogen peroxide (H_2_O_2_) for 10 min to deactivate endogenous peroxidases, then heated in 10 mM citrate buffer pH 6.0 at 121 °C for 30 min. Finally, they were blocked with 5% bovine serum albumin (BSA) in Tris-buffered saline. Each slide was treated with anti-TGF-β1 mouse monoclonal antibodies (sc-130348, Lot#J3019, Santa Cruz Biotechnology, Dallas, TX, USA), anti-α-SMA mouse monoclonal antibodies (sc-53142, Lot#K1914, Santa Cruz Biotechnology, Dallas, TX, USA), and anti-NF-kB p65 rabbit polyclonal antibodies (phospho S536, Abcam, Cambridge, MA, USA) and incubated overnight at 4 °C. Following a thorough washing in PBS buffer the next day, the sections were subjected to incubation with secondary antibodies. The samples were incubated at room temperature for 30 min, then rinsed in PBS buffer and exposed to streptavidine–peroxidase complex for 20 min (Agilent Dako, Carpinteria, CA, USA). The samples were subsequently washed with PBS, and the reaction was seen using 3,3′-diaminobenzidine (DAB) chromogen from Agilent Dako, CA, USA. Prior to mounting and microscopic examination, the slides were counterstained with hematoxylin. Positive expression of TGF-β1, α-SMA, and NF-kB was observed as brown staining in liver sections. Tissue pictures were acquired by examination of slides using an Olympus DP71 optical microscope. The amount of TGF-β1, α-SMA, and NF-kB in individual sections was quantified using ImageJ analysis software (Fiji ImageJ (2.16.0) software, http://imagej.net/software/fiji/ (accessed on 9 May 2026)) (National Institutes of Health, Bethesda, MD, USA) in ten representative non-overlapping fields at a magnification of 200. The percentage of stained areas (quantification of IHC staining) was reported as the mean ± SEM [37].

### 2.11. Determination of Inflammatory Markers

Cytokine concentrations in liver homogenates were quantified using ELISA kits as per the instructions of the manufacturer. Small portions of liver (1 gm) were homogenized in 5 mL to 10 mL of phosphate-buffered saline (PBS) using a glass homogenizer placed on ice. Elabscience^®^ Mouse IL-1β (Cat# E-EL-M003), IL-4 (Cat# E-EL-M0043), IL-5 (Cat# E-EL-M0722), and IL-10 (Cat# E-EL-M0046) are available. TGF-β1 (Elabscience^®^ Mouse, Cat#E-EL-M0051). The research used the methodology provided by the manufacturer, Elabscience Biotechnology Co., Ltd. (14780 Memorial Drive, Suite 105, Houston, TX 77079, USA). The studies were meticulously carried out utilizing a microplate reader (Spectra Max i3X, Molecular Devices, San Jose, CA, USA). Each measurement was repeated three times to ensure precision and consistency. Cytokine concentrations were normalized to total protein content, measured by the Bradford assay.

### 2.12. Determination of mRNA Expression of Hepatic α-SMA, MMP9, TNF-α, IL-6, NF-kB-p65 Genes

A quantitative real-time PCR approach was used to quantify the transcription levels of fibrotic markers such as α-SMA, MMP9, and inflammatory markers TNF-α, IL-6, and NF-kB-p65. The isolation of total RNA from 30 mg of mouse liver tissue was performed using TRizol reagent (15596-026, Life Technologies, Carlsbad, CA, USA) following the instructions provided by the manufacturer. A spectrophotometer (Thermo Scientific, Multiskan GO, East Lyme, CT, USA) was used to quantify the purity and concentrations of the RNAs collected. One μg of total RNA was used to produce complementary DNA (cDNA) using the iScript cDNA synthesis kit from Bio-Rad in Hercules, CA, USA. The cDNA samples were stored at a temperature of sub −20 °C until the subsequent tests. The gene expression profiles were evaluated using the SYBR Green-based qPCR technique from Bio-Rad in Hercules, CA, USA, in a Rotor-Gene Q instrument from Qiagen in Hilden, Germany. Table 1 provides a comprehensive summary of the primers utilized in the investigation. Each primer set was created using the Primer 3 web application. The developed primers were further validated in silico for target specificity using the Basic Local Alignment Search Tool (BLAST) https://ftp.ncbi.nlm.nih.gov/blast/db (accessed on 9 May 2026) [38]. The reactions were conducted under the indicated conditions: An initial denaturation step was conducted at 94 °C for 15 min, followed by 40 cycles of denaturation at 94 °C for 15 s, annealing at 60 °C for 30 s, and extension at 94 °C for 1 min, and final extension for 1 min at 94 °C. The 2^−ΔΔCt^ comparative Ct method approach developed by Livak and Schmittgen was used for the relative measurement of gene expression [39]. Normalization and relative quantification were performed using β-actin.

### 2.13. Statistical Analysis

Results were analyzed in GraphPad Prism Version 9 (Graphpad Software Inc., San Diego, CA, USA) and expressed as means ± SD. Normality was assessed by the Kolmogorov–Smirnov test. For parametric analysis, one-way analyses of variance (ANOVA) followed by Tukey’s post hoc analysis were used to determine any statistical differences between groups. For nonparametric analysis, including granuloma size and histological scores, the Kruskal–Wallis comparison test followed by post hoc pairwise comparison was used to determine any statistical differences between treated groups. Data were considered significant if the *p*-value was <0.05.

## 3. Results

### 3.1. Combination Therapy Reduces Worm and Egg Burden in S. mansoni-Infected Mice

**Figure 2:**
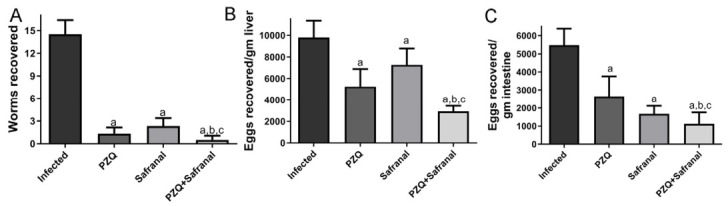
The effects of PZQ, safranal, and PZQ + safranal on the worms and eggs recovered from the liver and intestine of mice infected with *S. mansoni*. (**A**) Worms recovered. (**B**) Eggs recovered from the liver; the number of eggs in the liver was determined as eggs per gm. (**C**) Eggs recovered from the intestine; the number of eggs in the intestine was determined as eggs per gm. All results are expressed as means ± SD (n = 6). Significance was determined by one-way ANOVA test followed by Tukey’s post hoc analysis. ^a^ *p* < 0.05, significant change with respect to the infected group; ^b^ *p* < 0.05, significant change with respect to the PZQ group, significant change compared to the safranal group; ^c^ *p* < 0.05.

Eleven weeks after infection, untreated mice harbored a mean of 14.7 ± 1.3 adult worms (Figure 2A). PZQ monotherapy significantly reduced worm burden by 90.9% (*p* < 0.05 vs. infected). Safranal alone also significantly reduced worm count (by 84.1%), and the combination of PZQ + safranal showed the best reduction (96.6%, *p* < 0.05 vs. infected; Figure 2A). Correspondingly, hepatic and intestinal egg loads were markedly decreased in treated groups, with the greatest reduction observed in the mice receiving the PZQ + safranal combination (Figure 2B,C). PZQ alone reduced liver egg count by 42.8% and intestinal egg count by 51.7% relative to infected controls. The PZQ + safranal combination achieved further reductions (67.3% in liver and 79.7% in intestine, *p* < 0.05 vs. infected). Notably, egg burdens in the combination group were significantly lower than with PZQ alone (Figure 2B,C). These data indicate that adding safranal to PZQ significantly improves the clearance of *S. mansoni* adults and eggs compared with either monotherapy, suggesting an enhanced, possibly additive, effect of the combination.

### 3.2. Combination Therapy Improves Liver Histology and Reduces Granuloma Size

**Figure 3:**
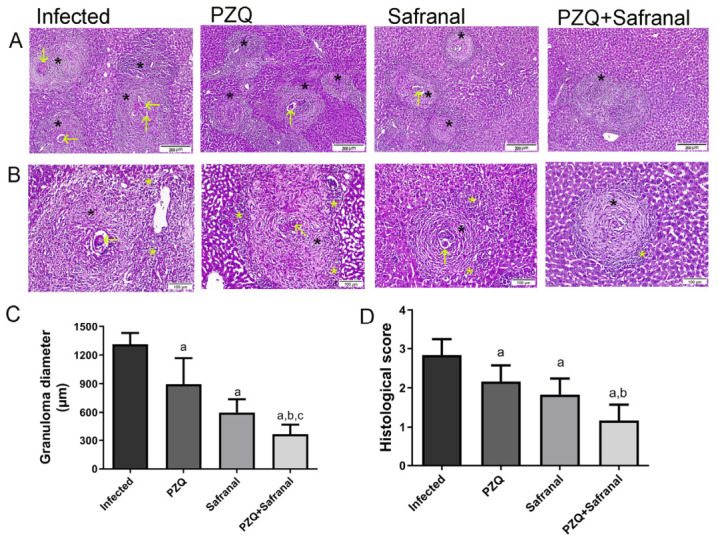
PZQ and safranal combination therapy improves liver histology and granuloma size of mice infected with *S. mansoni*. (**A**) A representative H&E-stained histological image of the liver after 11 weeks of infection in drug-untreated and drug-treated mice. The photographs were taken at 10×. (**B**) The photographs were obtained at a magnification of 20×. Black stars represent granulomas, yellow stars show infiltrating leukocytes around granulomatous lesions, and a yellow arrow indicates schistosome eggs. The liver from the infected group showed huge, widespread zones of coagulative necrosis with substantial inflammatory cell infiltration, whereas the liver from the PZQ and safranal groups showed smaller granulomas and low hepatic necrosis. (**C**) Granuloma diameter. The average sizes of 36 granulomas measured in liver sections from six infected mice each (6 granulomas per mouse) are displayed. (**D**) Histological score of the liver histology image. Quantification or scoring was performed on six slides in each group. 10 random microscopic fields were examined on each slide. The data are expressed as means ± SD (n = 6). The nonparametric Kruskal–Wallis H test was used to establish significance, which was then followed by a post hoc pairwise comparison. ^a^ *p* < 0.05 indicates a significant change compared to the infected group; ^b^ *p* < 0.05 indicates a significant change compared to the PZQ group. ^c^ *p* < 0.05 compared with the safranal group.

Histological analysis of H&E-stained liver sections revealed prominent egg-induced granulomas in infected controls (Figure 3A,B). In infected livers, granulomas contained central schistosome eggs surrounded by proliferating fibroblasts and heavy inflammatory cell infiltrates. Large zones of coagulative hepatocyte necrosis and leukocyte infiltration were evident around granulomas. Treatment with PZQ or safranal alone markedly reduced these pathological features, with granulomas appearing smaller and peripheral liver necrosis largely diminished (Figure 3A,B). The most pronounced improvement was seen with combined therapy, where mice treated with PZQ + safranal had the smallest granulomas and minimal peri-granulomatous damage (Figure 3A,B). Quantitative image analysis confirmed these observations; the average granuloma diameter was significantly reduced in all treated groups compared to infected controls, and granulomas in the combination group were significantly smaller than in the PZQ-only group (Figure 3C). Likewise, histological injury scores were significantly lower in the combination-treated mice than in infected or PZQ-only mice (Figure 3D). These results demonstrate that combined PZQ and safranal treatment substantially improves liver histopathology in schistosomiasis.

### 3.3. Combination Therapy Reduces Hepatomegaly and Liver Enzyme Elevations

**Figure 4:**
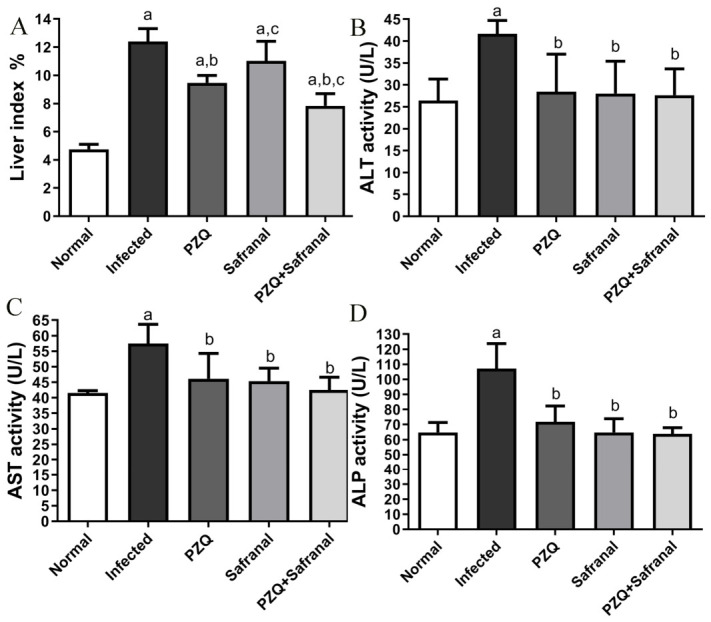
PZQ and safranal combination therapy improves liver index and reduces liver damage of *S. mansoni*-infected mice. (**A**) Liver index. (**B**) ALT, (**C**) AST, and (**D**) ALP activities measured in serum as markers of liver damage. All results are expressed as the means ± SD (n = 6). Significance was determined by one-way ANOVA test followed by Tukey’s post hoc analysis. ^a^ *p* < 0.05, significant change with respect to the control group; ^b^ *p* < 0.05, significant change compared to the infected group, ^c^ *p* < 0.05 significant change with respect to the PZQ group.

Chronic *S. mansoni* infection for 11 weeks caused significant hepatomegaly, as indicated by a marked increase in the liver index (liver weight/body weight) compared to controls (Figure 3A). Treatment with PZQ, safranal, or both significantly reduced this hepatomegaly. The greatest decrease in liver index was observed with the combined therapy (Figure 4A), although the difference between treated groups was not statistically significant. Consistent with liver injury, serum ALT, AST, and ALP activities were markedly elevated in infected mice (Figure 4B–D). Administration of PZQ, safranal, or both significantly lowered ALT, AST, and ALP levels toward normal (Figure 3B–D). Again, the combination therapy showed the most pronounced reductions in enzyme levels, although the additional decrease compared to monotherapy was not statistically significant. Together, these findings indicate that safranal enhances the hepatoprotective effects of PZQ, mitigating enzyme markers of liver damage in schistosomiasis.

### 3.4. Combination Therapy More Effectively Resolves Liver Fibrosis

**Figure 5:**
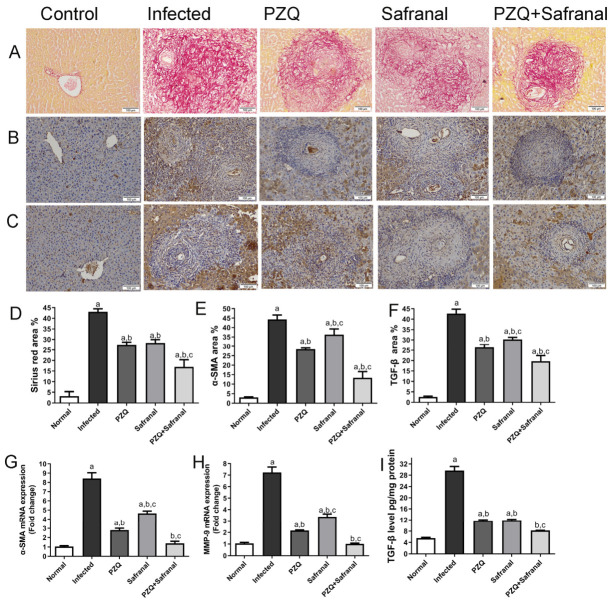
PZQ–safranal combination is better than monotherapy in resolving liver fibrosis in *S. mansoni*-infected mice. (**A**) Representative images of Sirius Red staining on liver sections of the mice (magnified ×200). Red indicates the areas of collagen deposition. (**B**) Representative images of the expression of α-SMA detected by immunohistochemistry (magnified ×200). (**C**) Representative images of the expression of TGF-β detected by immunohistochemistry (magnified ×200). (**D**) Quantification of Sirius Red staining areas. The fibrotic area, as a percentage of the total area, was quantified by ImageJ software (n = 6 for each group). (**E**) The positive staining area of α-SMA, as a percentage of the total area, was quantified by ImageJ software (n = 6 for each group). (**F**) The positive staining area of TGF-β as a percentage of the total area. (**G**–**I**) Gene expressions of α-SMA, MMP-9 and TGF-β in the liver. RNA transcription levels of fibrotic markers, including α-SMA and MMP-9, measured by qPCR. Data values are represented as means ± SD (*n* = 6). Significance was determined by one-way ANOVA test, followed by Tukey’s post hoc analysis. ^a^ *p*-value < 0.05 compared to the control group; ^b^ *p*-value < 0.05 compared to the infected group; ^c^ *p*-value < 0.05 compared with the PZQ group.

Sirius Red staining revealed extensive collagen accumulation (red staining) in the livers of infected mice, whereas control livers showed minimal staining (Figure 5A,D). Treatment with either PZQ or safranal significantly reduced collagen deposition. Notably, combined PZQ + safranal therapy led to a further marked reduction in the collagen-positive area (Figure 5A,D). Immunohistochemistry confirmed these results; infected livers had strong α-SMA and TGF-β1 expression around granulomas (Figure 5B,C,E,F), while treated groups showed progressively less staining. The combination group exhibited the lowest α-SMA and TGF-β1 positivity among all groups (Figure 5B,C,E,F). At the molecular level, qPCR analysis showed that infected mice had significantly elevated hepatic mRNA expression of fibrotic markers (α-SMA, TGF-β1, and MMP-9) (Figure 5G–I). Treatment with either PZQ or safranal significantly downregulated these genes (*p* < 0.05 vs. infected), and the combination therapy produced the greatest suppression of fibrogenic gene expression (Figure 5G). These data indicate that combined therapy more effectively attenuates the fibrotic response in schistosomal livers compared with either treatment alone.

### 3.5. Combination Therapy Ameliorates Liver Oxidative Stress

**Figure 6:**
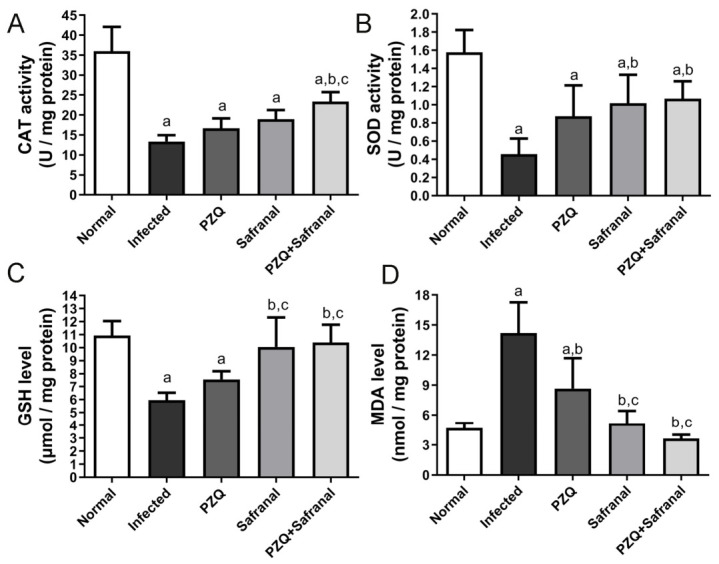
The effects of PZQ, safranal, and PZQ + Safranal treatment on the antioxidant and oxidative parameters of in the livers of *S. mansoni*-infected mice. (**A**–**D**) Oxidative stress markers assessed in liver homogenates include CAT, SOD, GSH, and MDA. The data values are the means ± SD (n = 6). Significance was determined by one-way ANOVA test, followed by Tukey’s post hoc analysis. ^a^ *p* < 0.05, significant change with respect to the control group; ^b^ *p* < 0.05, significant change compared to the infected group, and ^c^ *p* < 0.05 significant change compared to the PZQ group.

Schistosome infection caused profound oxidative stress in the liver, as infected mice showed a significant decrease in antioxidant defenses (catalase, superoxide dismutase, and glutathione) and a concomitant increase in MDA levels (Figure 6). Treatment with either PZQ or safranal significantly countered these changes, with each treatment elevating SOD, CAT, and GSH activities and reducing MDA compared to infected controls (Figure 6). Importantly, the PZQ + safranal combination had the most substantial effect on redox balance, where the mice receiving both drugs exhibited the highest antioxidant enzyme activities and the lowest MDA levels among all groups (Figure 6). These results demonstrate that safranal, when combined with PZQ, enhances the overall protection against oxidative stress and alleviates schistosomiasis-induced hepatic oxidative damage.

### 3.6. Combination Therapy Suppresses Inflammatory Responses

**Figure 7:**
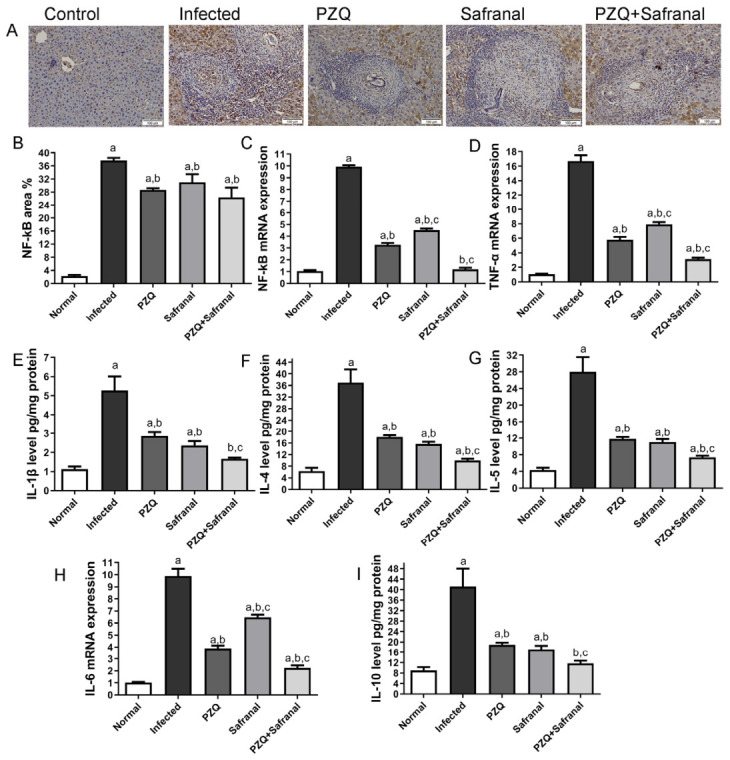
PZQ and safranal combination therapy reduces the inflammatory response in the livers of *S. mansoni*-infected mice. (**A**) This image shows representative immunohistochemistry for NF-κB in a mouse liver, magnified by 200×. PZQ and safranal therapy significantly reduces NF-κB area expression %. (**B**) Using ImageJ, the percentage of NF-κB positive regions in *S. mansoni* granulomas decreased dramatically after treatment with PZQ, safranal, or both. (**C**) NF-κB mRNA expression. (**D**–**I**) Cytokine concentrations in mouse liver homogenates following *Schistosoma mansoni* infection. (**D**) TNF-alpha mRNA, (**E**) IL-1 beta, IL-4 (**F**), IL-5 (**G**), IL-6 mRNA (**H**), and IL-10 (**I**) levels. We used qPCR to evaluate the RNA transcription levels of NF-κB, TNF-alpha, and IL-6 mRNA. The data values represent the means ± SD (n = 6). Significance was determined by one-way ANOVA test followed by Tukey’s post hoc analysis. ^a^ A *p*-value less than 0.05 suggests a comparison with the control group; ^b^ a *p*-value less than 0.05 shows a comparison with the infected group; and ^c^ a *p*-value less than 0.05 indicates a comparison with the PZQ group.

IHC staining for NF-κB (p65) showed intense nuclear localization in granuloma-associated cells of infected livers, indicating activation of this pro-inflammatory pathway (Figure 7A,B). Treatment with either PZQ or safranal alone significantly reduced NF-κB positive staining, while combined therapy further suppressed NF-κB activation (Figure 7B). Likewise, NF-κB (p65) mRNA expression was significantly upregulated in infected mice and was reduced by all treatments, most prominently following the combination therapy (Figure 7C). In parallel, infected mice had markedly elevated levels of proinflammatory cytokines TNF-α and IL-1β in liver tissue (Figure 7D,E), as well as elevated Th2 cytokines IL-4, IL-5, IL-6, and IL-10 (Figure 7F–I). In the praziquantel-treated group, these cytokine levels were significantly reduced, most likely as a consequence of schistosome elimination, while safranal monotherapy lowered them through its intrinsic anti-inflammatory and antioxidant properties (Figure 7D–I). Notably, the combined PZQ + safranal treatment produced the lowest levels of all examined cytokines and NF-κB (Figure 7D–I). These findings indicate that the combined therapy most effectively suppresses the hepatic inflammatory response (both Th1 and Th2) to schistosome infection.

## 4. Discussion

Current pharmaceutical treatment for schistosomiasis, which is almost exclusively based on PZQ, is quite successful in eliminating adult worms, but it is not very good at preventing reinfection or completely managing long-term liver damage and hepatic fibrosis. The only FDA-approved medication that is now widely used to treat human schistosome infections is PZQ. However, there are worries regarding its limited impact on fibrosis and disease transmission, as well as the potential for the parasite to become resistant in large-scale drug administration regimens [40,41]. In order to enhance schistosomiasis results, there is growing interest in creating additional and alternative therapy options, such as combination regimens and new medicines. The aim of this study was to assess the impact of combined PZQ and safranal treatment against the progression of *S. mansoni*-induced hepatic cellular damage and fibrosis. Our findings suggest that the observed reductions in liver index following administration of safranal, PZQ, and their combination are likely associated with a decrease in hepatic egg load, a reduction in granuloma count and size, and overall improvement in hepatic pathology. Moreover, PZQ or safranal and their combination reduced lobular inflammation and localized necrosis in histological sections compared to infected animals. Histological examination and assessment of liver injury markers revealed significant improvement in *S. mansoni*-infected mice treated with the combined PZQ and safranal protocol. Concomitant administration of both drugs resulted in a decreased egg load in the liver and small intestine and reduced granuloma size, suggesting a possible additive effect between the two drugs. We report a statistically significant reduction in worm count in a murine model following treatment with safranal alone or the PZQ–safranal combination, which enhanced the PZQ-induced reduction in hepatic egg load. These findings suggest that the PZQ–safranal combination may represent a possible additive strategy to address pathogenesis associated with *S. mansoni*.

Schistosomal infection induces oxidative damage in the liver [13,15]. MDA is the byproduct of lipid peroxidation in host cells and serves as a marker of cellular oxidative stress. Granuloma-inflammatory cells in *S. mansoni* infection produce superoxide and hydroxyl radicals, which help to produce lipid peroxides [42]. In the present investigation, hepatic MDA values increased significantly in infected mice. The current results demonstrated that safranal, either alone or combined with PZQ, dramatically reduced MDA levels. The safranal effect could be attributed to a reduction in egg burden, a major source of free radicals, or to its antioxidant properties [19,43]. The study also linked *S. mansoni* infection to a decline in antioxidant defense. Infected mice had significantly lower CAT and SOD activity, as well as glutathione levels. Infected animals treated with safranal and PZQ had significant reductions in MDA as well as increased antioxidant levels. Liver fibrosis is identified by changes in its ECM [44]. Activating quiescent HSCs produces myofibroblast-like cells with enhanced proliferation, ECM buildup, and expression of α-smooth muscle actin (α-SMA) [44]. Schistosomiasis is associated with liver fibrosis because parasite eggs and the host’s immune response coexist. Fibrosis is described as the buildup of collagen in the liver [44,45]. HSC activation causes hepatic fibrogenesis, with α-SMA as the most used marker [6,7]. Chronic liver injury activates HSCs, which express α-SMA and produce ECM [6,7]. Our investigation found α-SMA expression in liver tissue, particularly around granulomas. There is evidence that HSCs may be involved in *S. mansoni* -induced liver fibrosis. TGF-β, a fibrogenic cytokine, activates both HSCs and fibroblasts [45,46]. TGF-β is generated by many cell types in the liver and promotes HSC activation in injured liver tissue [46]. The presence of TGF-β in infected liver tissue suggests that HSCs and TGF-β are important contributors to the progression of hepatic fibrosis induced by schistosomiasis. The present investigation revealed a notable increase in collagen fibers within the infected liver. However, the administration of PZQ, safranal, and a combination of PZQ and safranal decreased collagen deposition. When administered together, PZQ and safranal markedly reduced collagen deposition and the expression of fibrotic markers such as α-SMA and TGF-β, compared with either treatment alone. Furthermore, infected mice exhibited increased hepatic expression of matrix metalloproteinase-9 (MMP-9). Both safranal and the combined PZQ–safranal treatment significantly reduced MMP-9 expression, indicating potential anti-fibrotic effects. Notably, TGF-β and TNF-α are known to induce MMP-9 activity during hepatic fibrogenesis [12], thereby amplifying extracellular matrix remodeling and inflammatory signaling [47]. Moreover, MMP-9 plays a key role in early events in neutrophil recruitment cascades and transmigration in liver injury models, and its reduction leads to diminished neutrophil migration [48]. Collectively, these findings suggest that the combination of PZQ and safranal may represent an additive therapeutic strategy for mitigating schistosomiasis-associated liver fibrosis in mice. While the present study employed comprehensive immunohistochemical and molecular markers (including Sirius Red staining, α-SMA, TGF-β, and fibrosis-related gene expression) to evaluate hepatic fibrosis, we acknowledge that direct biochemical quantification of collagen content, such as a hydroxyproline assay, would provide additional quantitative confirmation. Furthermore, future studies assessing the expression of collagen-specific genes (e.g., Col1a1 and Col3a1) may further strengthen the mechanistic understanding of the antifibrotic effects of safranal, particularly in combination with PZQ.

Inflammation plays a critical role in the development and progression of hepatic fibrosis [49]. During schistosome infection, the liver and intestines exhibit multi-cellular granulomatous inflammation surrounding the deposited eggs [1,5]. Granulomatous inflammation activates HSCs, promoting their proliferation and migration toward the periphery of egg granulomas [1,5]. In the present study, untreated infected mice showed elevated hepatic levels of IL-1β, TNF-α, IL-4, IL-5, IL-6, and IL-10, indicating concurrent Th1 and Th2 immune responses. Multiple cytokines coordinate the initiation, progression and resolution of hepatic granuloma formation and fibrosis [5]. Mice infected with *S. mansoni* exhibit elevated levels of both Th1 cytokines (IL-1β, TNF-α) and Th2 cytokines (IL-4, IL-5, IL-6, IL-10) in serum and liver tissues [11,50], likely reflecting the robust inflammatory response triggered by egg deposition. TNF-α, a pleiotropic cytokine, activates many signaling pathways implicated in liver fibrosis [51]. Pro-inflammatory cytokines such as TNF-α and IL-1β activate NF-κB signaling, thereby promoting the survival and persistence of activated HSCs [52]. Furthermore, Th2 cytokines, including IL-4, IL-5, and IL-10, contribute substantially to granuloma formation, collagen deposition, and the progression of fibrosis [53]. Our study demonstrated that safranal significantly reduced the levels of IL-1β, TNF-α, IL-4, IL-5, IL-6, and IL-10. Consistent with Gupta et al. [18], safranal has been shown to suppress TNF-α, IL-6, and IL-1β, thereby exerting anti-inflammatory effects. We have previously reported that safranal also exhibited anti-inflammatory activity in a liver cancer model [43]. Consistently, combined treatment with PZQ and safranal reduced IL-1β, TNF-α, IL-4, IL-5, IL-6, and IL-10 levels, inhibited granuloma growth, and attenuated liver fibrosis. Taken together, these findings support the hypothesis that the PZQ–safranal combination exerts an additive anti-inflammatory effect that may contribute to the mitigation of hepatic granulomatous inflammation and fibrosis.

NF-κB is a key regulator of inflammation, and its activation is critical for the release of pro-inflammatory cytokines in response to pathogen-associated molecular patterns. NF-κB plays a dual role in inflammation, as it is involved in both the initiation and regulation of inflammatory responses [54]. Activation of NF-κB signaling modulates the activity of hepatocytes, HSCs, and Kupffer cells, thereby contributing to the progression of hepatic fibrosis [55]. Indeed, hepatic granuloma formation and fibrosis are closely associated with NF-κB activation [56,57]. In the present study, we observed increased NF-κB immunopositive brown staining and elevated gene expression in liver tissues that correlated with infection and fibrosis progression. Treatment with either PZQ or safranal alone produced only modest reductions in NF-κB immunostaining and gene expression, whereas the combined treatment significantly decreased NF-κB levels. Consistently, previous work has shown that safranal pre-treatment reduces microbial ligand (LPS)-induced IL-6 and TNF-α levels, while LPS + ATP increases IL-1β production [23]. Moreover, safranal pre-treatment prior to LPS priming inhibits NF-κB-p65 phosphorylation, thereby reducing the production of pro-inflammatory cytokines (IL-6 and TNF-α) and pro-IL-1β expression. Taken together, the presented findings suggest that modulation of NF-κB signaling may underlie the anti-inflammatory and anti-fibrotic effects observed with the combination therapy.

**Figure 8:**
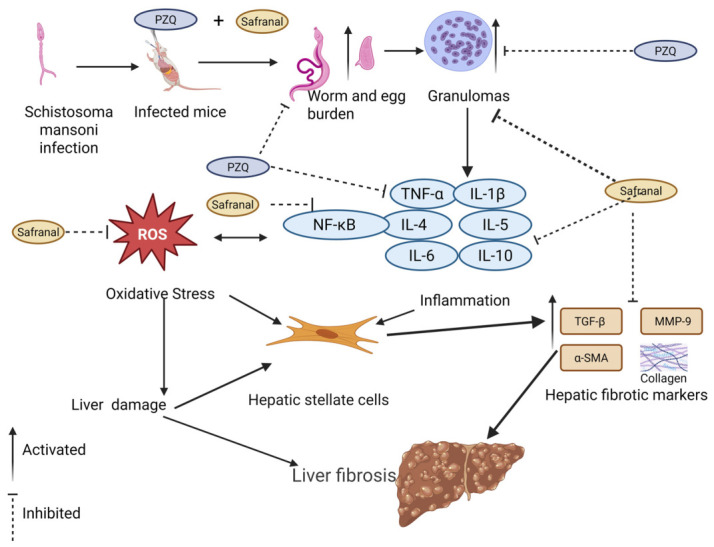
Graphical summary depicting the hepatic pathology induced by *Schistosoma mansoni* infection and the protective effects of combined PZQ and safranal treatment, highlighting diminished worm and egg burden, mitigation of granulomatous inflammation and oxidative stress, downregulation of pro-inflammatory cytokines and fibrotic markers, and overall enhancement of liver architecture. Created in BioRender, BioRender. https://BioRender.com/87l89ij (accessed on 9 May 2026).

Numerous natural products have been investigated as supplements or substitutes to PZQ in the treatment of schistosomiasis, specifically addressing liver damage, oxidative stress, and fibrosis. Natural substances such as rutin, wogonin, berberine, eugenol, and schisandrin B have been documented to mitigate Schistosoma-induced hepatic damage by regulating oxidative stress, inflammatory mediators, and fibrogenic pathways [11,32,58]. This work identifies safranal as a promising natural chemical with hepatoprotective, antifibrotic, and immunomodulatory properties in schistosomiasis. Safranal demonstrated antioxidant activity, indicated by elevated levels of GSH, CAT, and SOD, alongside decreased MDA, as well as a reduction in inflammatory mediators and profibrotic indicators (e.g., collagen deposition, α-SMA, MMP-9, and TGF-β) (Figure 8). These findings align with the antioxidant and anti-inflammatory properties attributed to safranal in various models of chemically induced liver injury, where it mitigated oxidative stress and reduced pro-inflammatory cytokines [18,19,43]. Safranal therapy has been demonstrated to be efficacious in a diethylnitrosamine (DEN)-driven hepatocellular cancer murine model, significantly diminishing all evaluated oxidative stress markers and reinstating antioxidant levels to baseline values [43]. In the DEN-induced HCC model pretreated with safranal, a significant reduction in inflammatory mediators was detected, including reduced expression of COX-2, iNOS, NF-κB, TNF-α, and its receptor pTNF-R1 [43]. Moreover, in a non-alcoholic fatty liver disease model, safranal administration reduced hepatic oxidative stress and correlated with diminished levels of cytokines and collagen in hepatocytes, hence reinforcing its antifibrotic and hepatoprotective properties [59].

## 5. Conclusions

The present investigation highlights the limitations of conventional pharmacological therapy for schistosomiasis, including the emergence of fibrosis and the progressive deterioration of liver function associated with the development of drug resistance. The aim of this study was to assess the efficacy of PZQ and safranal in managing liver fibrosis induced by *Schistosoma mansoni*. The findings suggest that the combined therapy exerts an additive effect in reducing hepatic egg burden, granuloma size, and liver fibrosis. The combined treatment also enhances antioxidant defenses, reduces oxidative stress, and decreases fibrotic markers such as α-SMA and TGF-β. Furthermore, the findings emphasize the anti-inflammatory properties of the PZQ–safranal formulation, as evidenced by the reduction of pro-inflammatory signaling associated with liver inflammation and fibrosis. These data suggest that the combined use of PZQ and safranal may represent a viable strategy to address the issue of PZQ resistance and more effectively control schistosomiasis-associated liver fibrosis (Figure 8). Our conclusions regarding the PZQ + safranal interaction remain preliminary and should be confirmed by future dose–response studies designed specifically to distinguish additive from synergistic effects. Therefore, further studies are necessary to validate these findings.

## Figures and Tables

**Table 1:** Primer sequences, target genes, for SYBR green rt-PCR.

Gene Name	Primer Pairs (5′–3′)	Genbank Accession
β-actin	Forward GTGACGTTGACATCCGTAAAGA	NM_007393
	Reverse GCCGGACTCATCGTACTCC	
α-SMA	Forward CCCAGACATCAGGGAGTAATGG	NM_007392
	Reverse TCTATCGGATACTTCAGCGTCA	
TNF-α	Forward CAGGCGGTGCCTATGTCTC	NM_013693
	Reverse CGATCACCCCGAAGTTCAGTAG	
MMP9	Forward GCAGAGGCATACTTGTACCG	NM_013599
	Reverse TGATGTTATGATGGTCCCACTTG	
NFkB-p65	Forward 5′-GGACAGCACCACCTACGATG-3′	NM_009045.4
	Reverse 5′ CTGGATCACTTCAATGGCCTC-3′	
IL-6	Forward 5′-GACAAAGCCAGAGTCCTTCAGA-3′	NM_001314054.1
	Reverse 5′-TGTGACTCCAGCTTATCTCTTGG-3′	

## Data Availability

The original contributions presented in this study are included in the article/Supplementary Materials. Further inquiries can be directed to the corresponding authors.

## Supplementary Materials

The following supporting information can be downloaded at https://www.mdpi.com/article/10.3390/jox16040120/s1. Figure S1: Original, unprocessed images corresponding to Figure 3 in the main text; Figure S2: Original, unprocessed images corresponding to Figure 5 in the main text; Figure S3: Original, unprocessed images corresponding to Figure 7 in the main text.
