# Supplementary material for: Safranal Enhances the Efficacy of Praziquantel Against Schistosoma mansoni Infection and Alleviates Liver Fibrosis, Inflammation and Oxidative Stress in Mice
Source: J Xenobiot. 2026 Jun 26;16(4):120. doi: 10.3390/jox16040120 (PMC13397992; doi:10.3390/jox16040120)
Supplement: Supplementary file 1 [file jox-16-00120-s001.zip › jox-4339085-supplementary.pdf]

Journal of Xenobiotics

# Supplementary Materials: Safranal Enhances the Efficacy of Praziquantel Against *Schistosoma mansoni* Infection and Alleviates Liver Fibrosis, Inflammation and Oxidative Stress in Mice

**Azza Fahmy, Amany Mohammed Mohmmed Hegab, Hanan S. Mossalem, Samah Sulaiman Abuzahrah, Saud Omar Alafghani, Alaaeldin Ahmed Hamza, Nouf Juaid and Amr Amin**

**Figure S1.** Original, unprocessed images corresponding to Figure 3 in the main text.

**A representative H&E-stained histological picture at 10×**

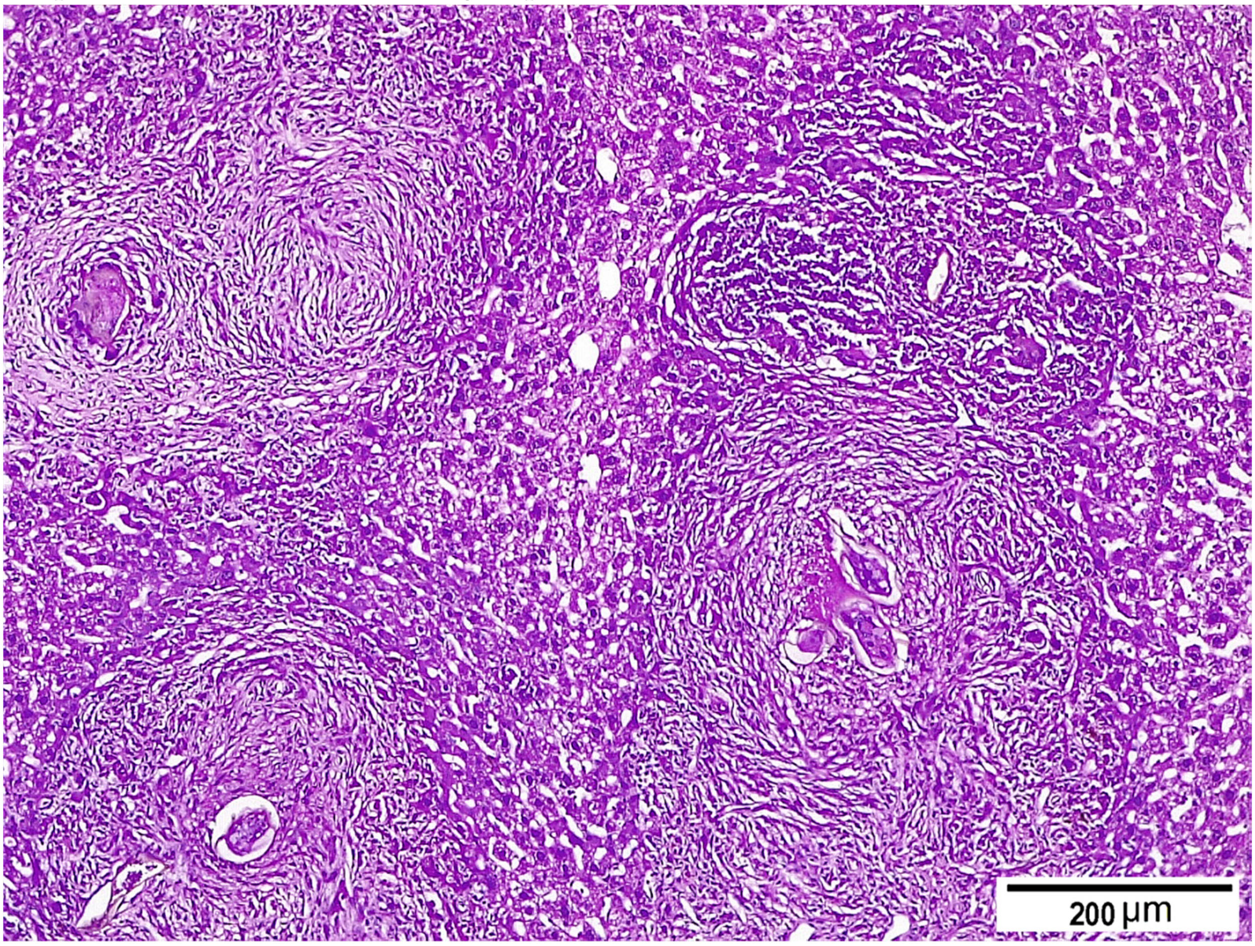


Infected 10×

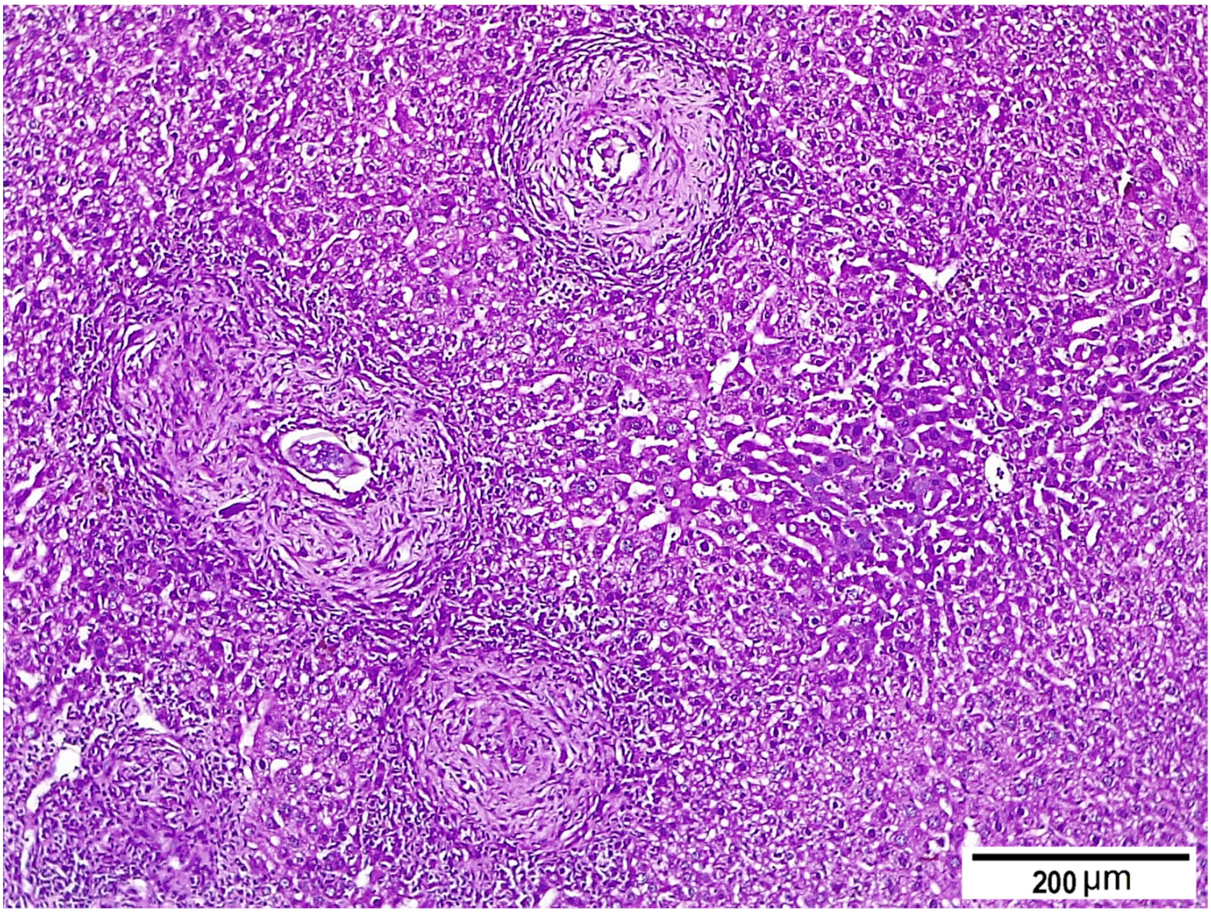


Safranal 10×

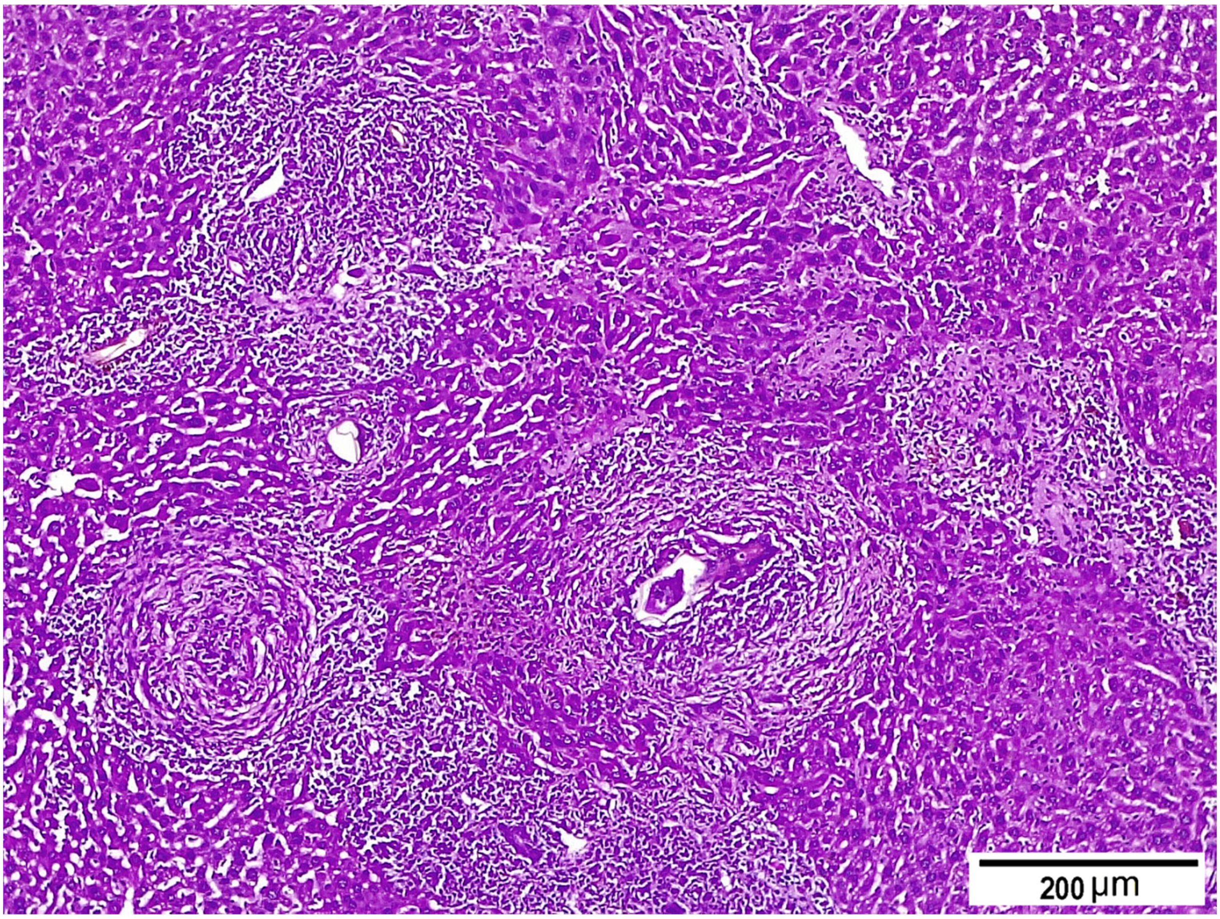


PZQ 10×

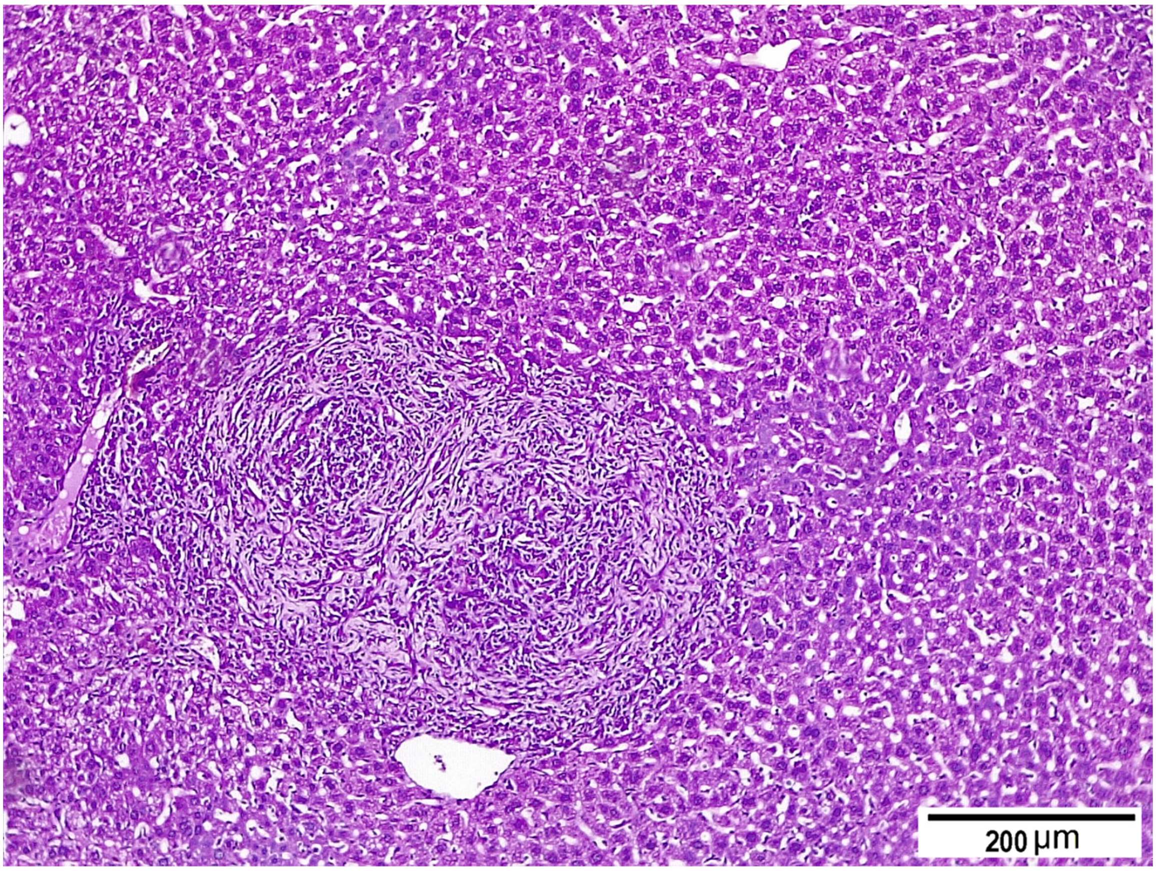


PZQ + Safranal 10×

**B representative H&E-stained histological picture at 20×**

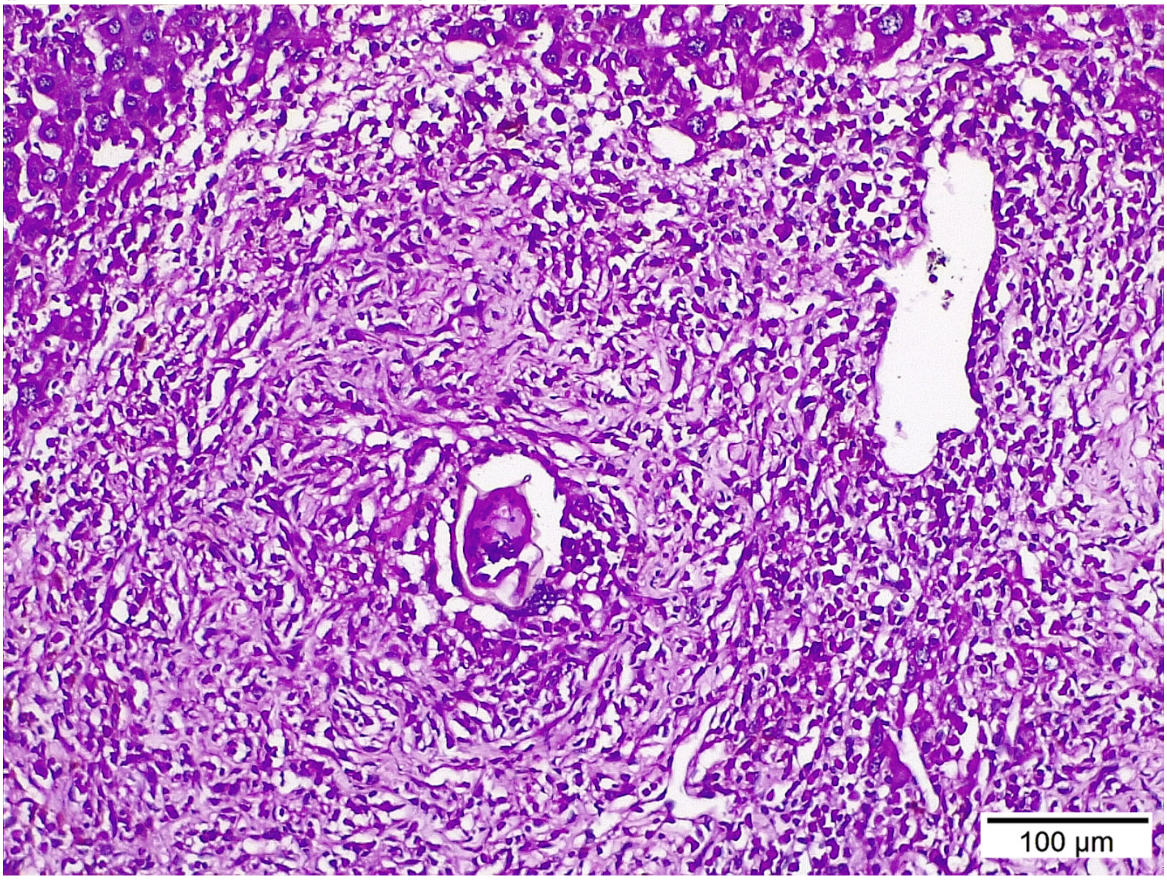


Infected 20×

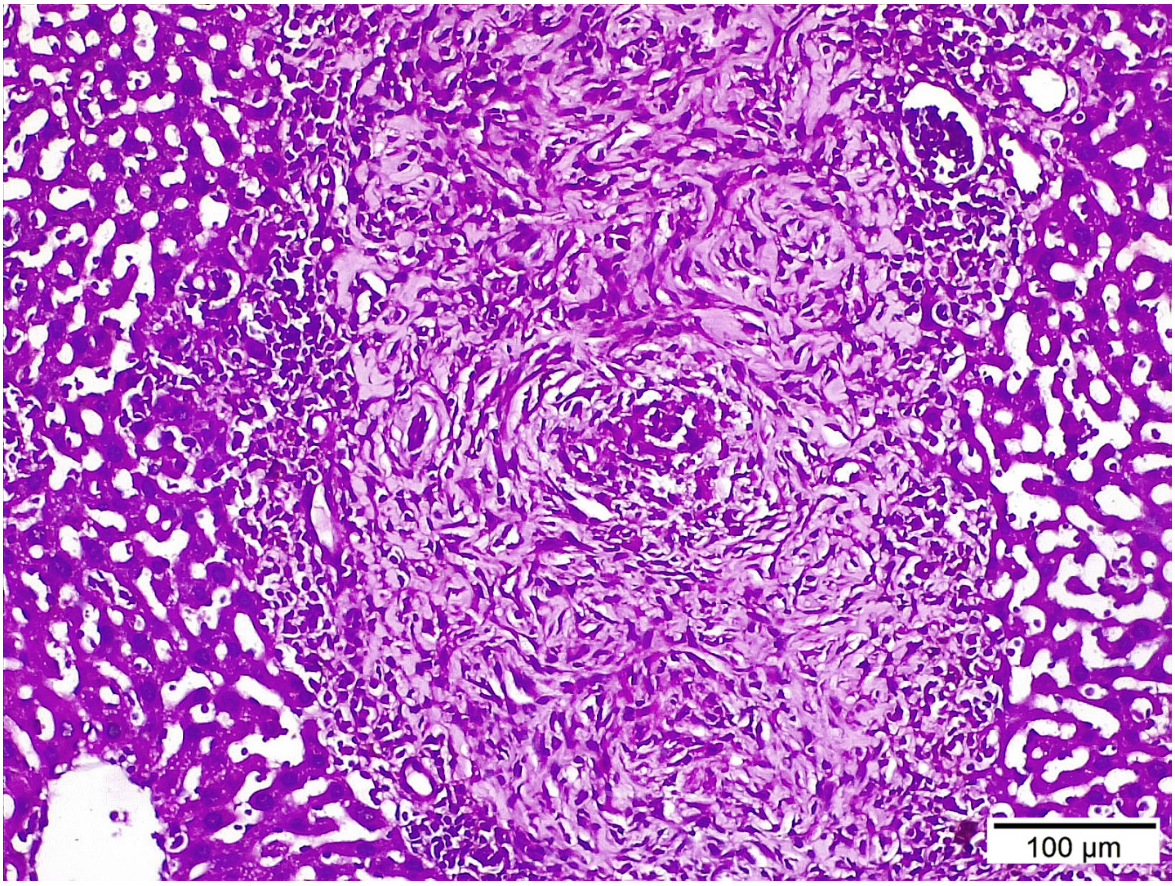

PZQ 20×

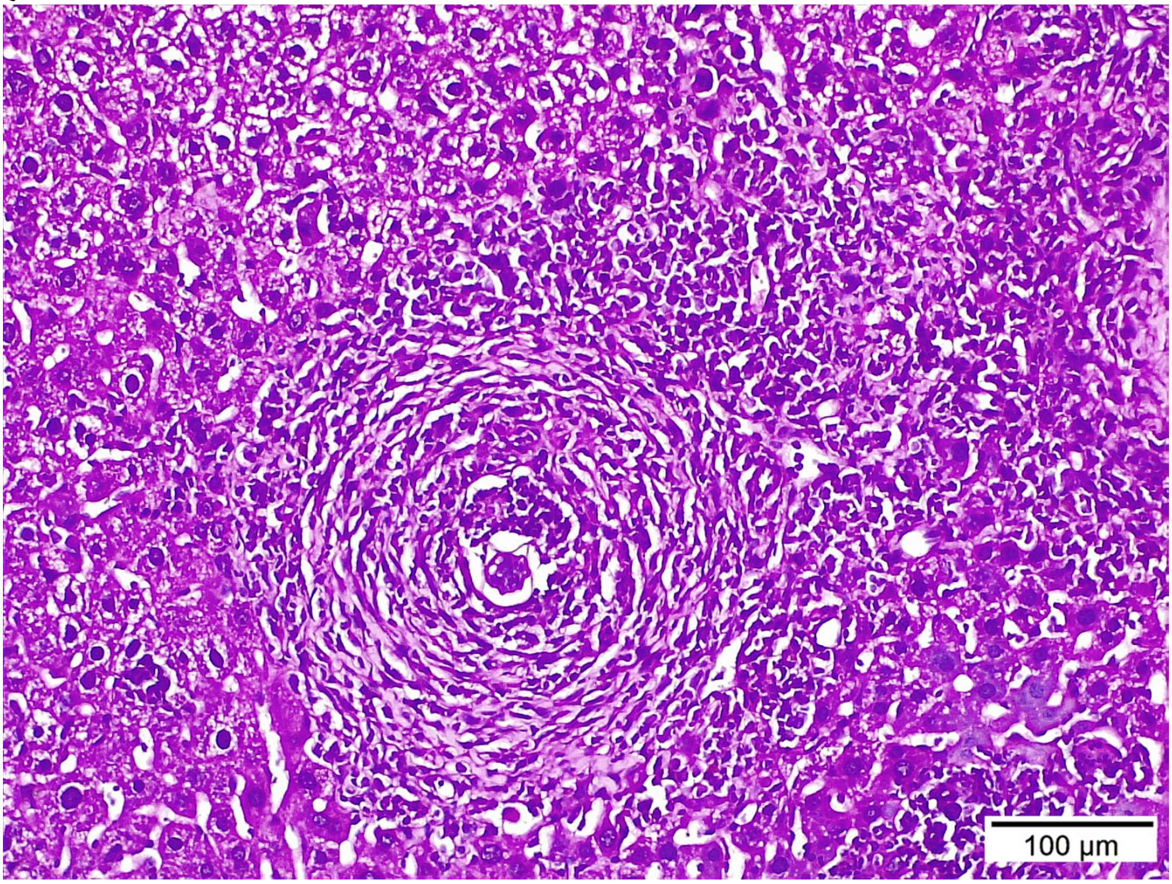

Safranal 20×

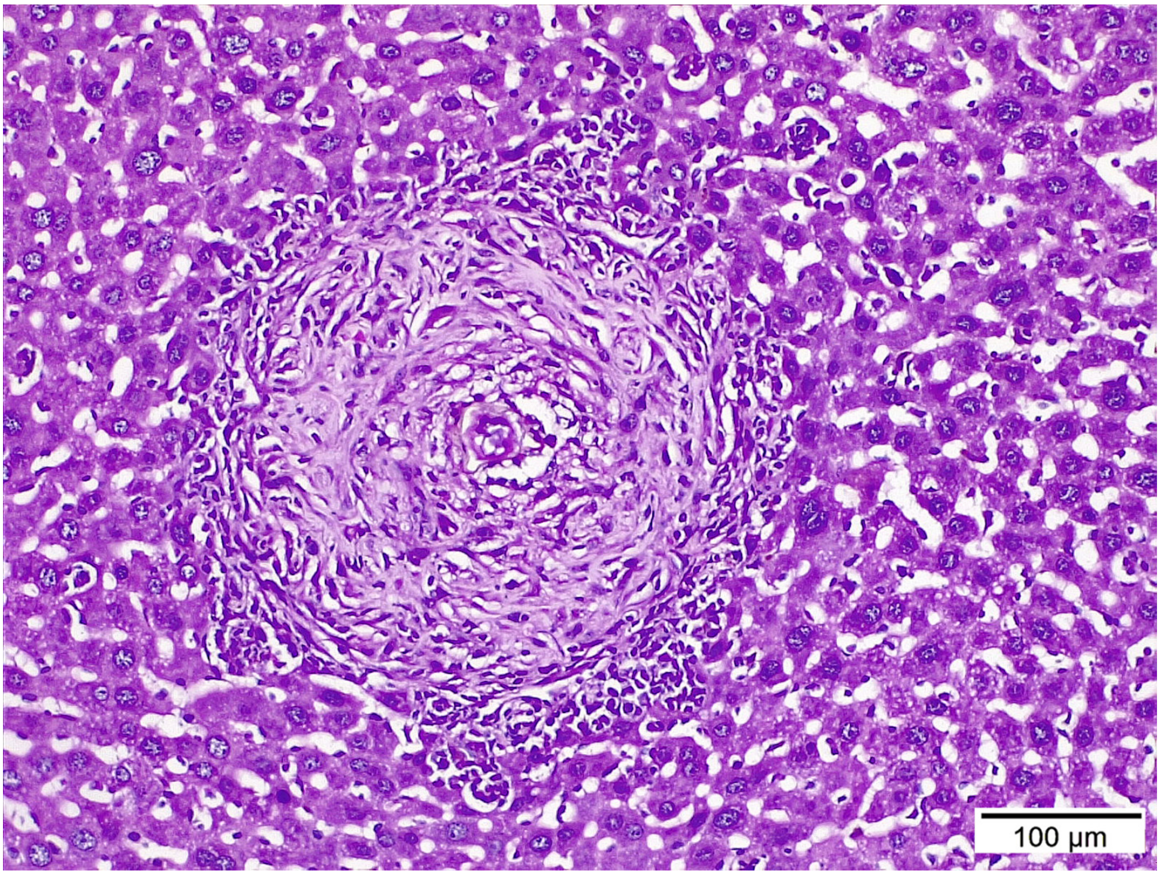


PZQ + Safranal 20×

**Figure S2.** Original, unprocessed images corresponding to Figure 5 in the main text.

(A) Representative images of Sirius red staining on liver sections of the mice (magnified × 200).

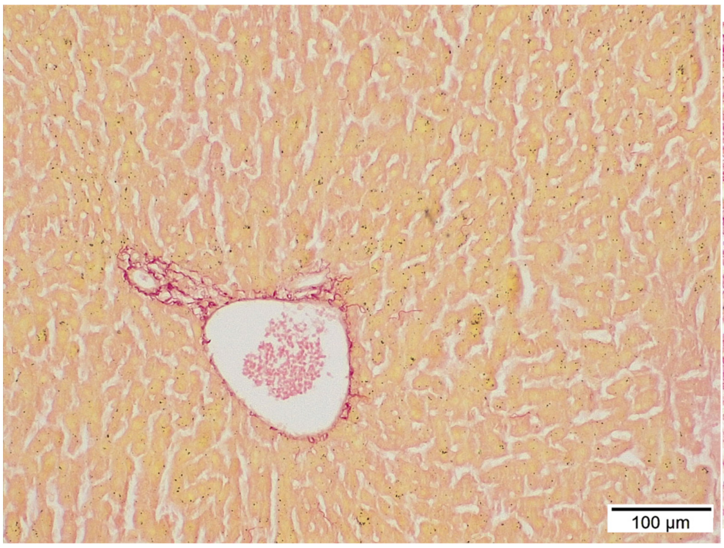


Control

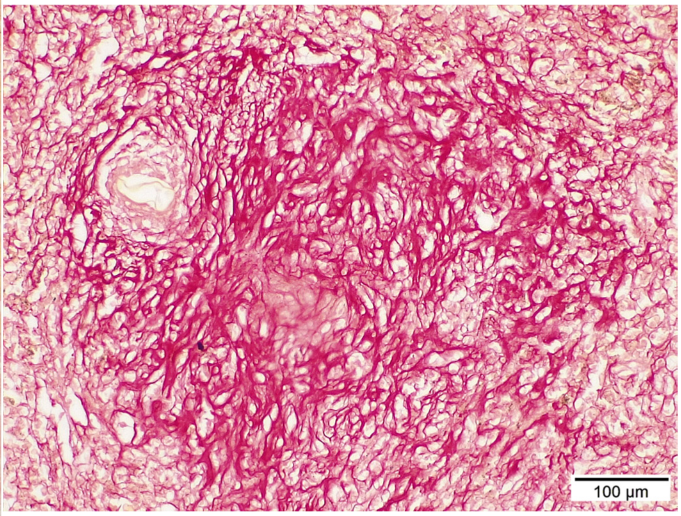


Infected

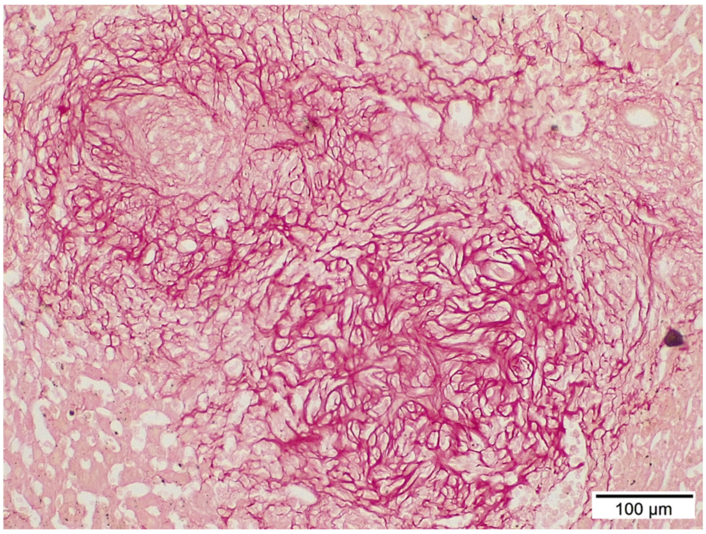


Safranal

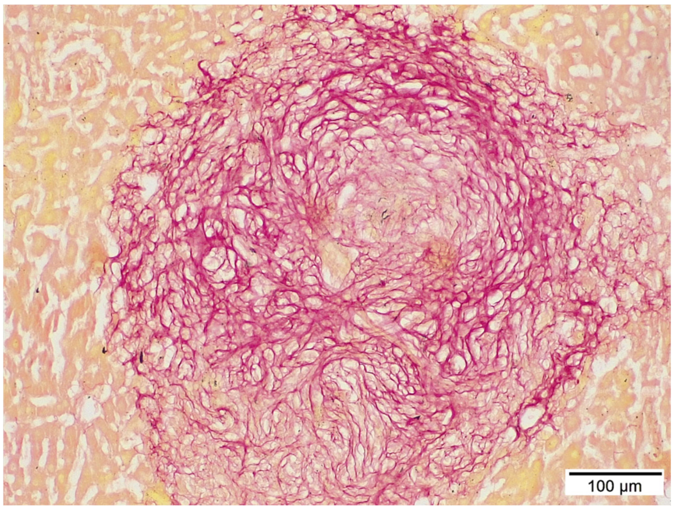


PZQ

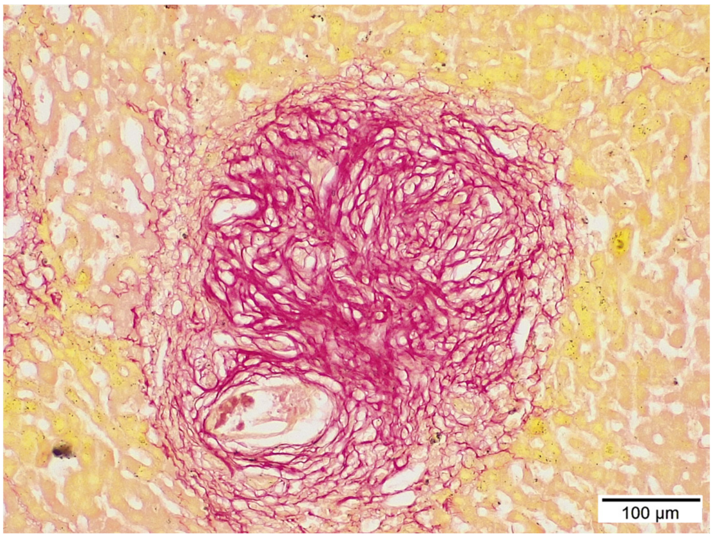


PZQ+Safranal

(**f**)

(B) Representative images of the expression of $\alpha$-SMA detected by immunohistochemistry (magnified × 200)

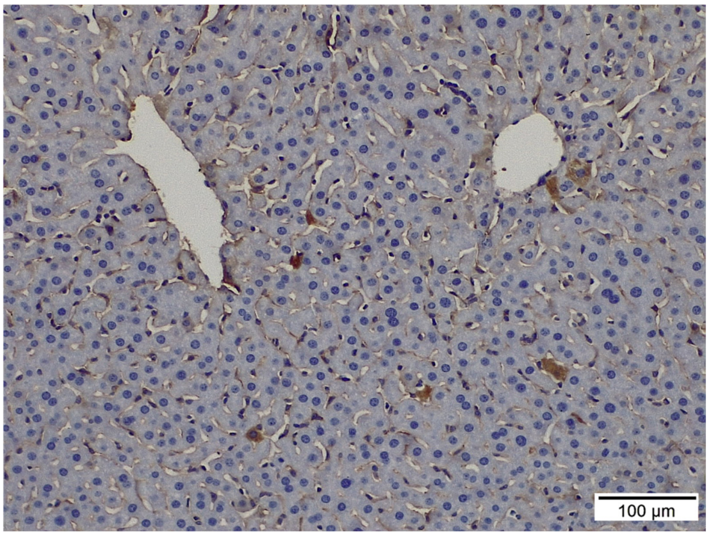


Control

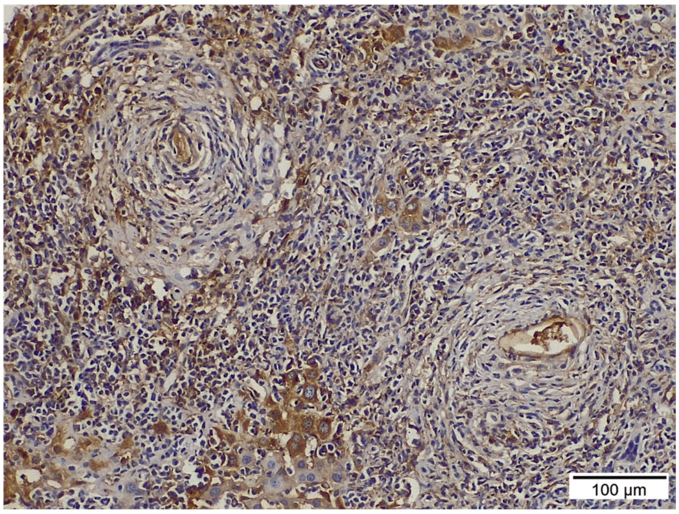


Infected

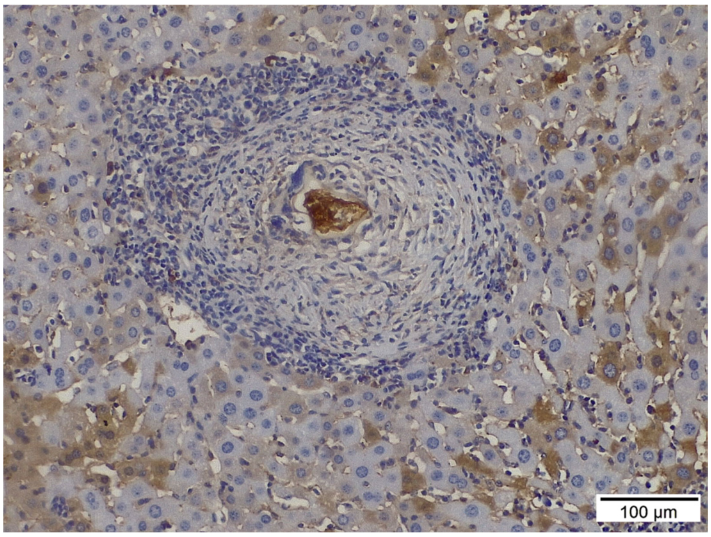


PZQ

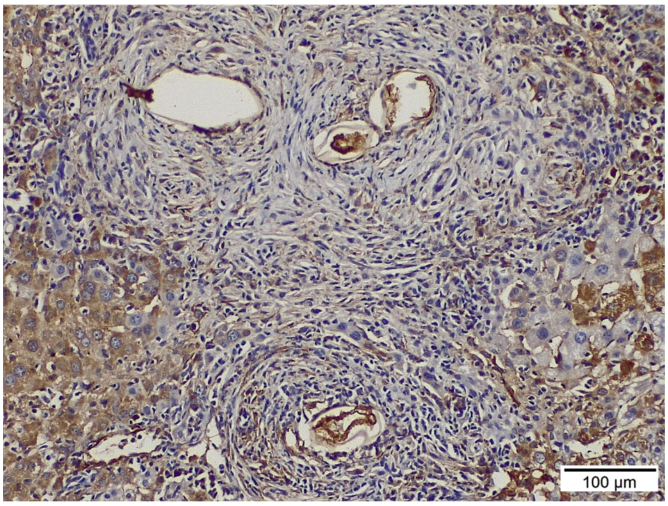


Safranal

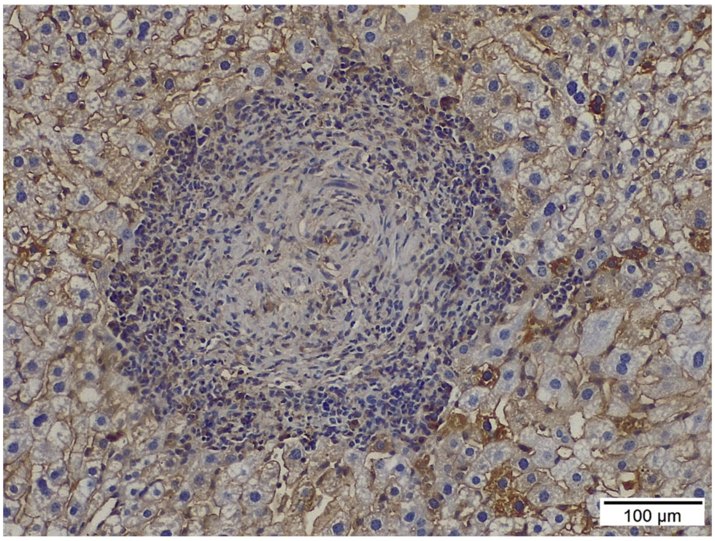


Safranal + PZQ

(**I**)

(C) Representative images of the expression of TGF-β detected by immunohistochemistry (magnified × 200)

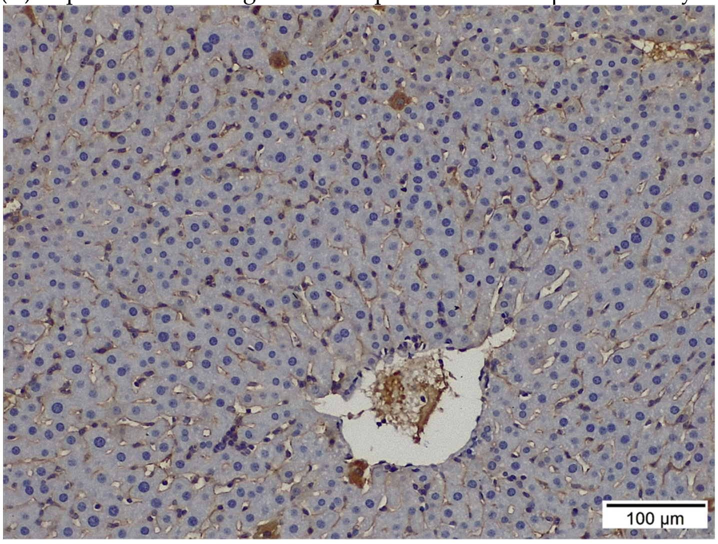


Control

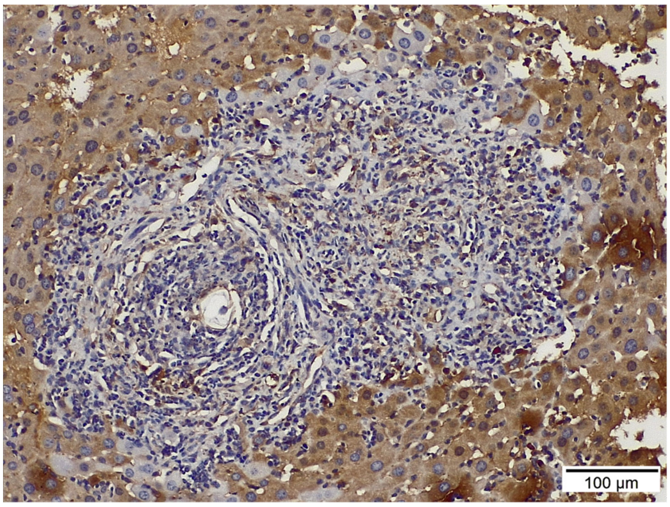


Infected

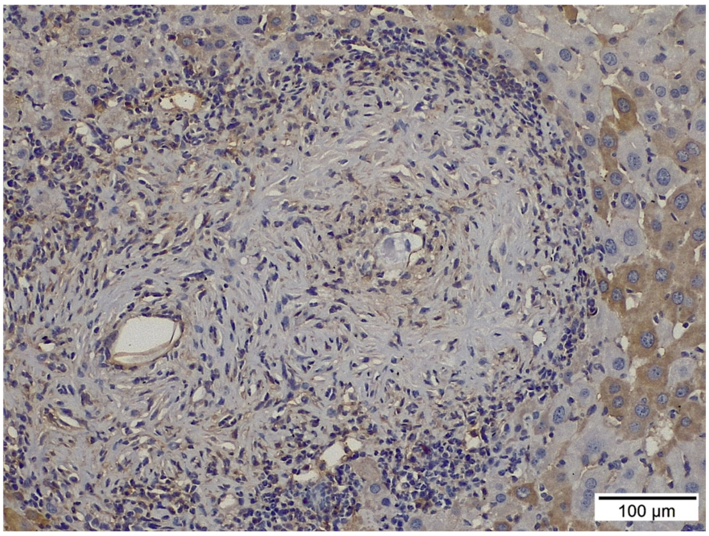

Safranal

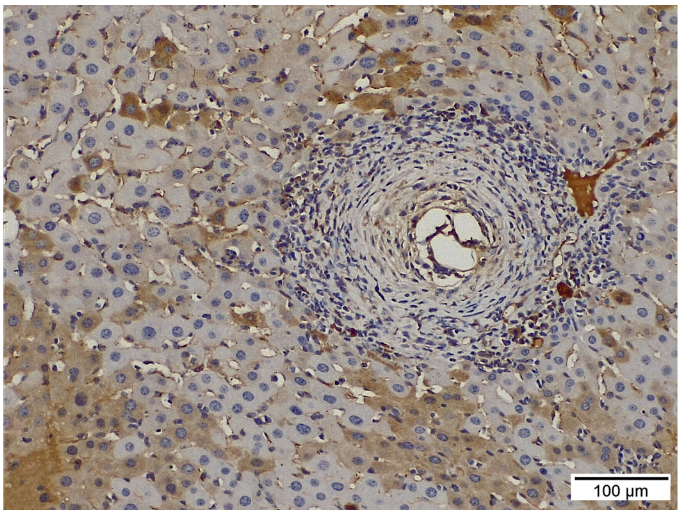

PZQ +Safranal

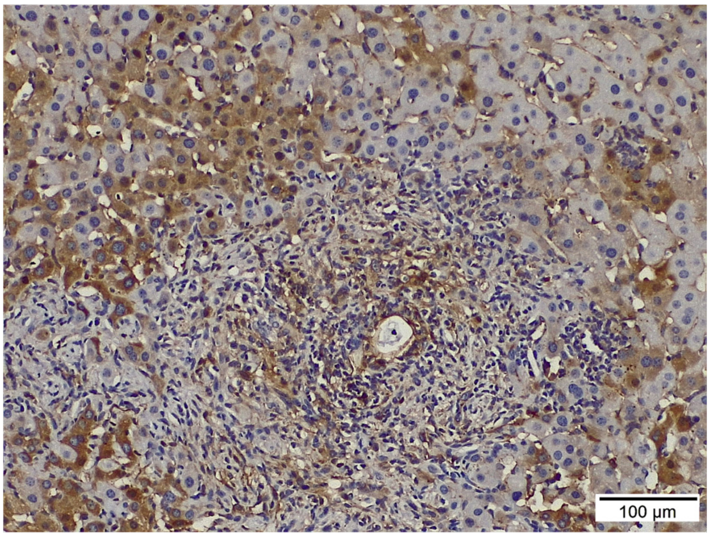

PZQ

**Figure S3.** Original, unprocessed images corresponding to Figure 7 in the main text.

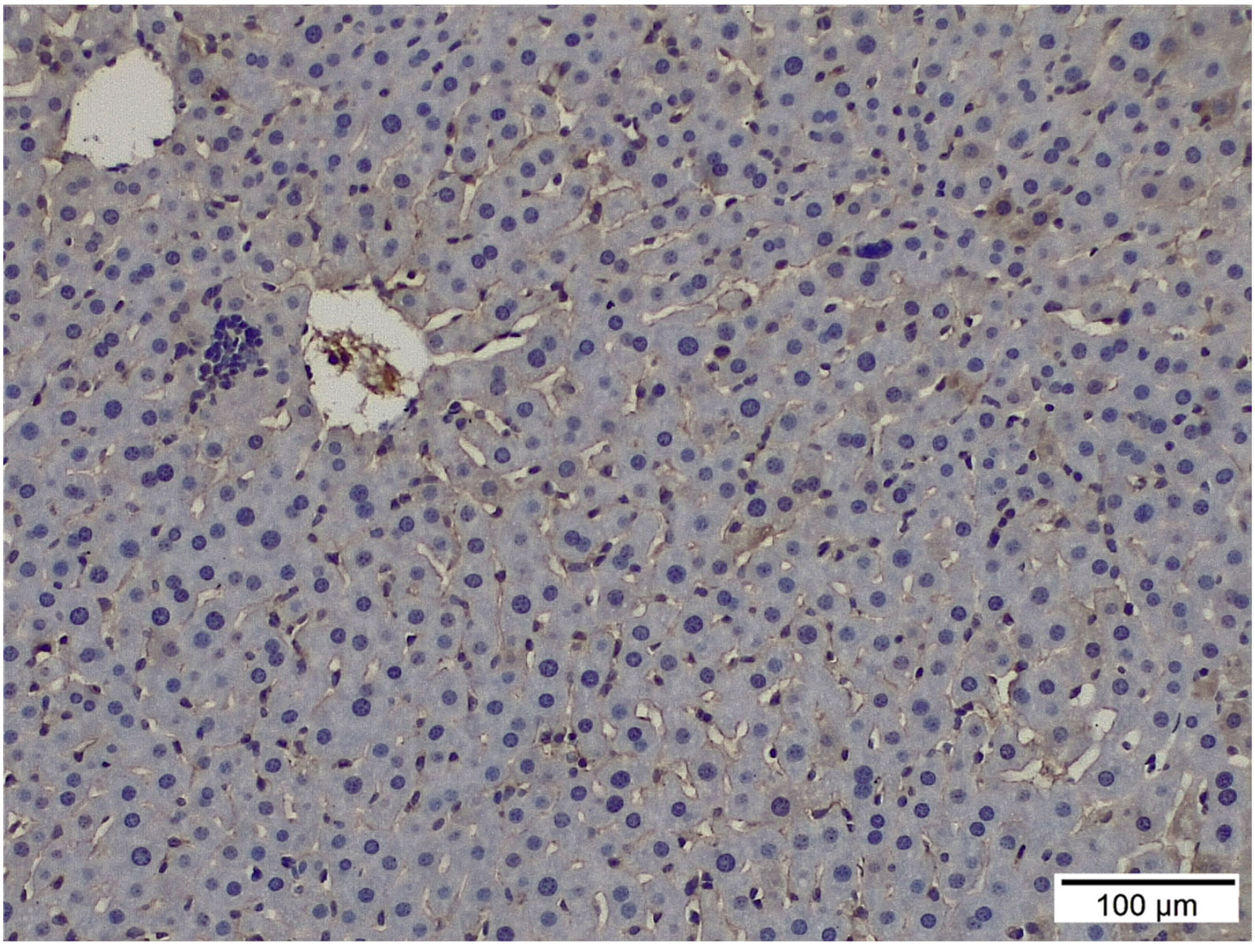


Control

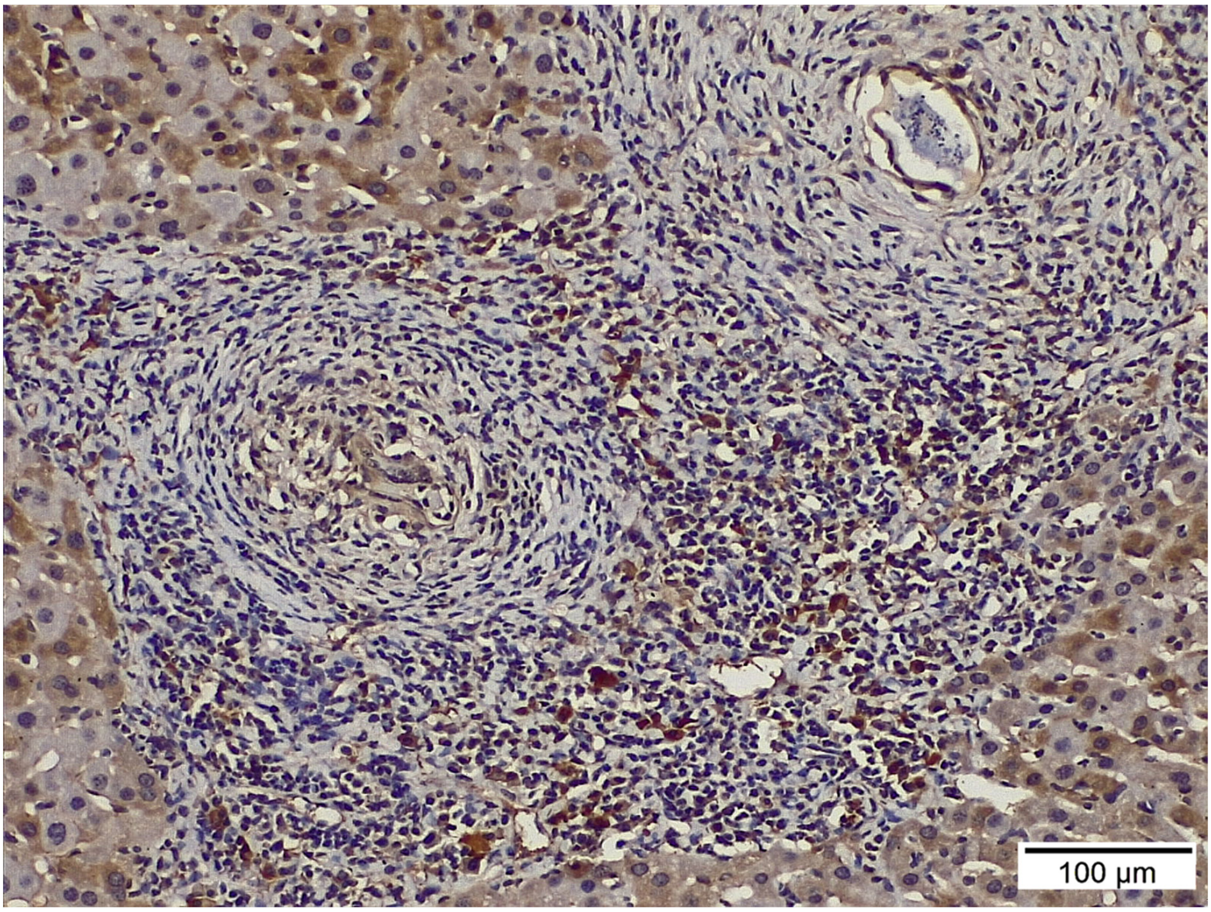


Infected

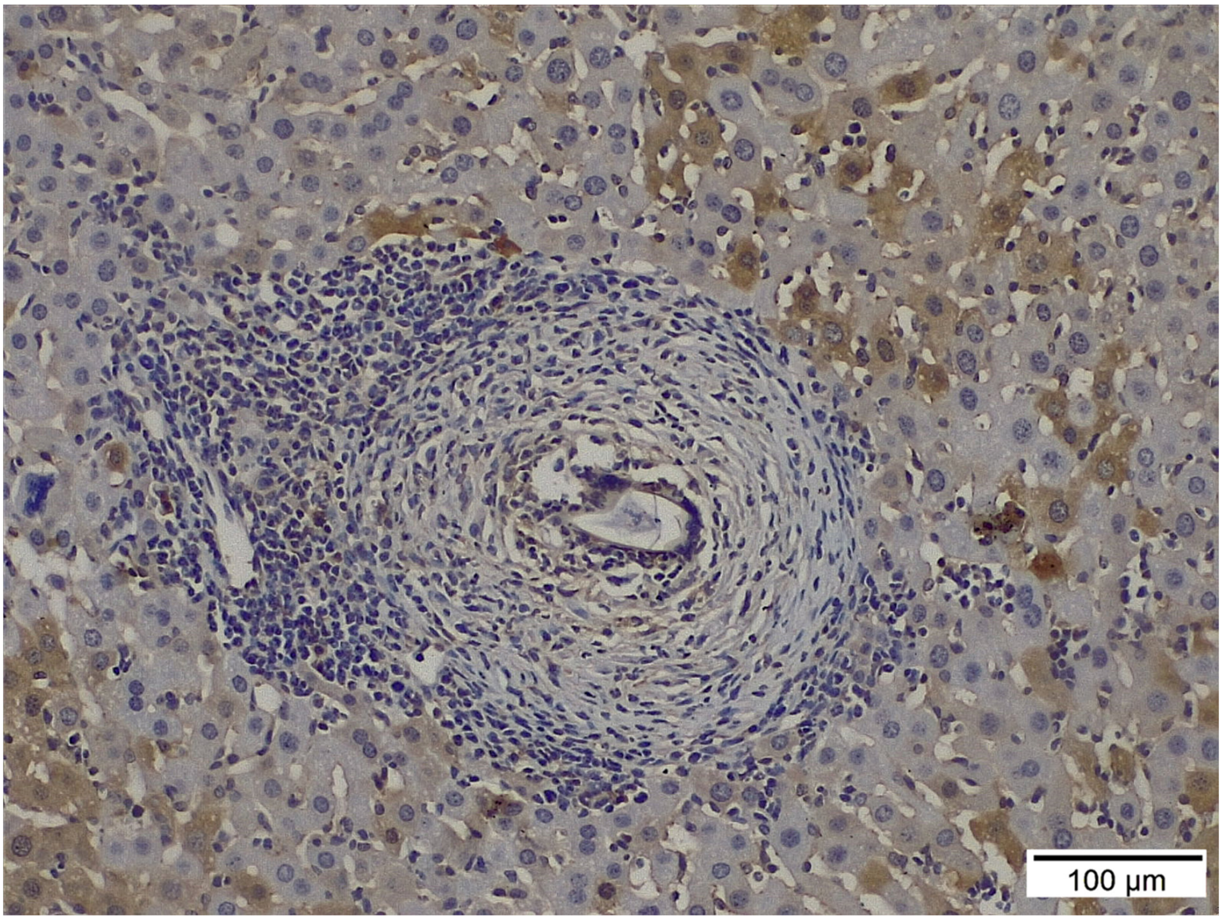


PZQ

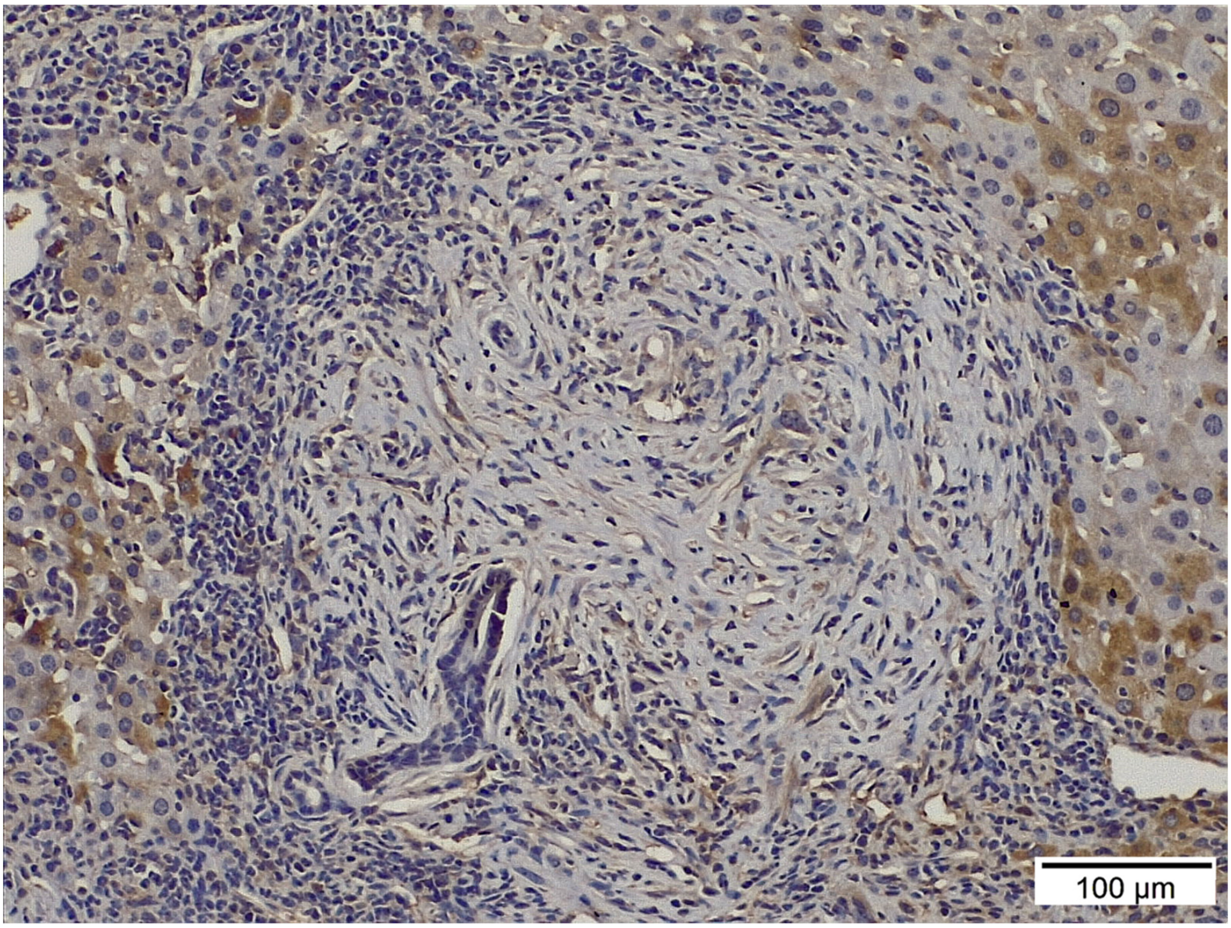

Safranal

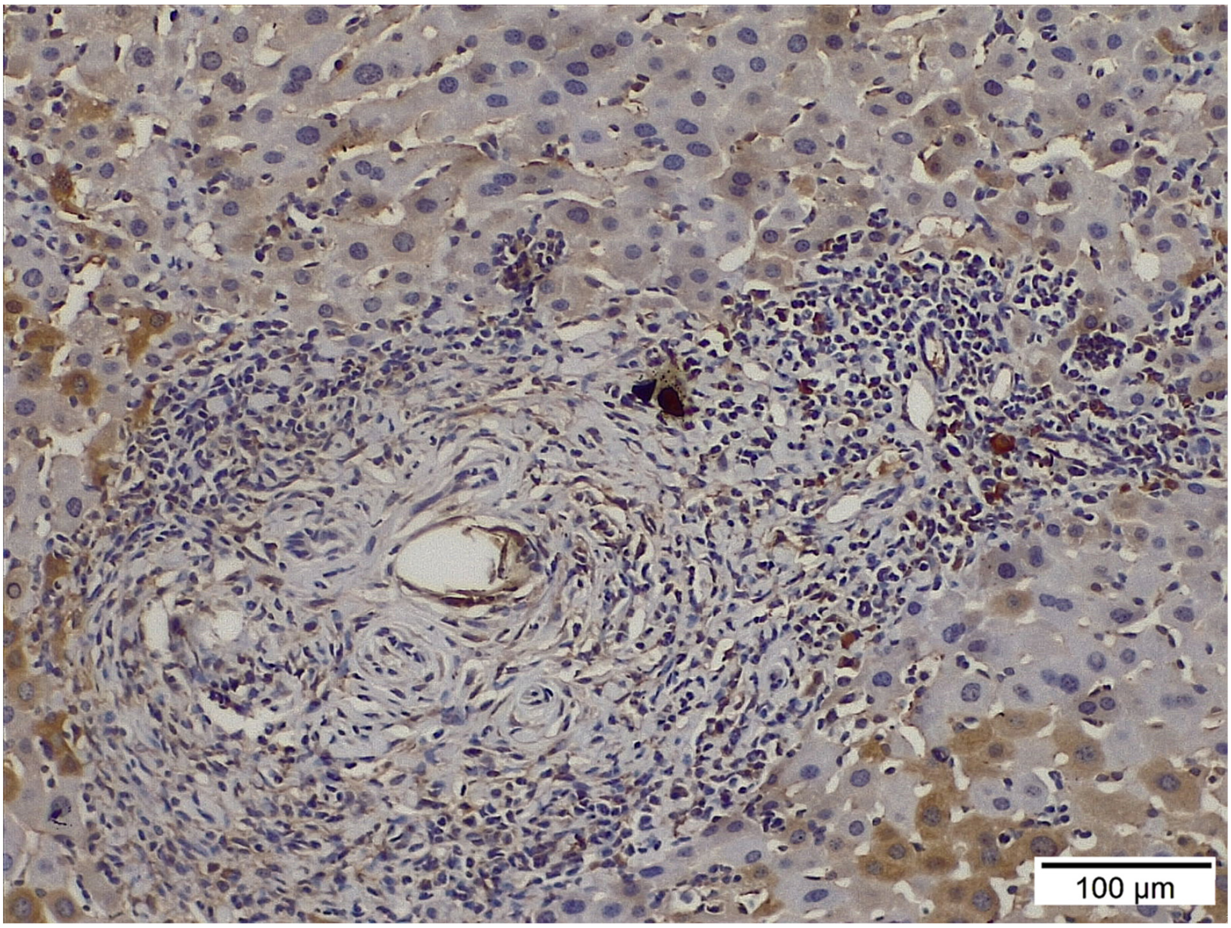

PZQ+Safranal
